# A longitudinal study of facial growth of Southern Chinese in Hong Kong: Comprehensive photogrammetric analyses

**DOI:** 10.1371/journal.pone.0186598

**Published:** 2017-10-20

**Authors:** Yi Feng Wen, Hai Ming Wong, Colman Patrick McGrath

**Affiliations:** 1 Paediatric Dentistry & Orthodontics, Faculty of Dentistry, The University of Hong Kong, Hong Kong SAR, China; 2 Periodontology & Public Health, Faculty of Dentistry, The University of Hong Kong, Hong Kong SAR, China; Seoul National University College of Medicine, REPUBLIC OF KOREA

## Abstract

**Introduction:**

Existing studies on facial growth were mostly cross-sectional in nature and only a limited number of facial measurements were investigated. The purposes of this study were to longitudinally investigate facial growth of Chinese in Hong Kong from 12 through 15 to 18 years of age and to compare the magnitude of growth changes between genders.

**Methods and findings:**

Standardized frontal and lateral facial photographs were taken from 266 (149 females and 117 males) and 265 (145 females and 120 males) participants, respectively, at all three age levels. Linear and angular measurements, profile inclinations, and proportion indices were recorded. Statistical analyses were performed to investigate growth changes of facial features. Comparisons were made between genders in terms of the magnitude of growth changes from ages 12 to 15, 15 to 18, and 12 to 18 years. For the overall face, all linear measurements increased significantly (p < 0.05) except for height of the lower profile in females (p = 0.069) and width of the face in males (p = 0.648). In both genders, the increase in height of eye fissure was around 10% (p < 0.001). There was significant decrease in nasofrontal angle (p < 0.001) and increase in nasofacial angle (p < 0.001) in both genders and these changes were larger in males. Vermilion-total upper lip height index remained stable in females (p = 0.770) but increased in males (p = 0.020). Nasofrontal angle (effect size: 0.55) and lower vermilion contour index (effect size: 0.59) demonstrated large magnitude of gender difference in the amount of growth changes from 12 to 18 years.

**Conclusions:**

Growth changes of facial features and gender differences in the magnitude of facial growth were determined. The findings may benefit different clinical specialties and other nonclinical fields where facial growth are of interest.

## Introduction

Successful restoration of harmonious facial esthetics is one of the primary goals of maxillofacial and plastic surgeries and orthodontic treatment. The relationships among various facial features can be modified by both growth and treatment. Knowledge of the amount and direction of growth changes therefore assist clinicians in planning the optimal treatment procedures [1]. Taking advantage of growth changes, the duration of orthodontic treatment could be shortened [2].

Growth changes of the facial soft tissues have been studied using various anthropometric approaches. Applying standardized anthropometric instruments, Farkas et al. performed a series of cross-sectional studies on facial growth of North American Caucasians [3–8]. While normative data were provided on a yearly basis from 1 to 18 years of age for various facial regions, there were altogether only 21 dimensions investigated and none of them were angular measurements or proportion indices. The direct anthropometric approach is time consuming and technique sensitive. In addition, there are inaccuracies inherent in this approach, such as soft tissue compressibility and sensitivity of the eyes [9].

Several longitudinal cephalometric studies have been undertaken to study growth of hard skeletal structures and soft tissue profiles based on samples from the Bolton study [10], Iowa facial growth study [11, 12], and Denver’s Child Research Council study [13]. However, most cephalometric studies focused only on parameters modifiable by orthodontic treatment [14]. In addition, Powell and Rayson [15] indicated that reliance on profile alone led to incomplete assessment of the overall face.

Recent technology advancement enabled researchers to acquire three-dimensional facial imaging data through instruments such as laser scanner [16], electromagnetic digitizer [17], and stereophotogrammetric system [18]. Sforza and colleagues investigated growth of the orbits [19], nose [17], lips [20], and ears [21] based on a sample of around 900 Italians aged 4 to 73 years. It is noteworthy that these studies, as well as the series of reports by Farkas et al. [3–8], are all cross-sectional investigations of facial growth. With cross-sectional data, it is difficult for one to determine whether the age-related changes are a reflection of real growth changes or are merely an artefact of changes in sample composition. Since growth changes are often subtle, it is estimated that at least twenty times as many participants as in longitudinal studies are required for cross-sectional studies to achieve the same level of accuracy in predicting incremental growth changes [22]. Longitudinal studies, on the other hand, provide more valid and accurate data [11] and are thus better suited for growth studies [23].

The advantages of two-dimensional photogrammetry has been mentioned previously [24, 25]. In summary, the method is free from radiation exposure, avoids the issue of soft tissue compressibility, and is time-saving. Besides, the equipment is portable and widely accessible. These characteristics make two-dimensional photogrammetry particularly suitable for field studies. With its excellent reliability, photogrammetry has been recommended as the optimal choice for large-scale epidemiological studies aiming at establishing population norms [24]. In spite of these advantages, a systematic review [26] indicated that there is currently a lack of longitudinal photogrammetric studies providing comprehensive assessment of facial growth. Although growth changes of 29 facial measurements between 4 and 13 years of age were reported by Bishara et al. [9, 27], the study has several significant limitations. First, there were only 20 participants recruited. The extremely small sample size rendered the study findings unrepresentative. Second, only linear facial measurements were analyzed. Angular and proportion facial parameters shed light on the relative positions of facial features. Failure to include these parameters impedes detailed understanding of facial structures. Third, the study findings were largely reported descriptively. Despite the authors’ claim about statistical analyses, significance of the longitudinal changes were not reported.

Age 12, 15, and 18 years represent three important developmental stages, corresponding to the late childhood, mid-adolescence, and early adulthood period, respectively. Based on a small sample of Caucasians, Nanda et al. [28] found active facial growth taking place from childhood through mid-adolescence to the early adulthood period. In addition, 12 to 18 years of age is a period when orthodontic treatment takes places. A thorough understanding of growth changes of facial features from 12 through 15 to 18 years of age is therefore of both research and clinical significance. The aim of the present study was to use serial photographic records of a population-representative sample of Chinese in Hong Kong to quantify changes in various facial parameters from 12 through 15 to 18 years of age.

## Participants and methods

### Study sample

This was a prospective longitudinal study conducted among a population-representative sample of Chinese in Hong Kong. To be eligible for inclusion, the participants had to satisfy the following criteria: (1) ethnic Chinese born in Hong Kong and originated from Guangdong province in southern China; (2) born during 1 April and 31 May 1997; (3) Class I molar and canine relationships. Participants were excluded if they: (1) demonstrated apparent facial disharmony (e.g., gross facial deformities, obvious facial asymmetry, severe maxillary and/or mandibular protrusion/retrusion, and incompetent lips); (2) had history of orthodontic therapy or maxillofacial surgery.

This study began in 2010. The sampling frame was all local secondary schools in Hong Kong (by law all children are required to attend secondary schools). A random sample of 45 schools (approximately 10% of all local secondary schools) from 18 districts in Hong Kong, SAR, was selected. The secondary schools were the primary sampling unit. Within each school all Form 1 and Form 2 (equivalent to US Grade 6 and 7) students born between 1 April and 31 May were invited to participate in the study. Parents/primary caregivers provided their written consent and students were asked to provide their assent. At 15 and 18 years of age, the participants were invited for a re-examination. The follow-ups took place between December 2012 and March 2013 and between June 2015 and September 2015.

Sample size calculation was performed based on the one-way repeated measures analysis of variance (ANOVA), a test suitable for longitudinal data analysis. Due to the lack of reports on effect size for longitudinal changes of facial parameters, an effect size of 0.06, which corresponds to the cut-off for low and medium magnitude of an effect [29], was used for sample size calculation. The selection of a low effect size for sample size calculation allowed us to detect small age-related changes in facial parameters. In addition, the selected effect size was close to the value of 0.077 reported in a recent study by Sforza et al. [30]. With a power of 0.90 and at a significance level of 0.05, it was estimated that 106 participants were needed for the analysis. Since all data analyses would be stratified by gender, a total of 212 participants with data from all three phases of this study were required. Anticipating 40% dropout rate in the first follow-up and 45% dropout rate in the second follow-up, 668 participants were recruited in 2010. Of these, 225 females and 211 males participated in the first follow-up and 215 females and 168 males participated in the second follow-up. There were 281 participants (160 females and 121 males) who participated in all three phases of this study. Among these 281 participants, 2 females were excluded due to apparent facial disharmony and another 4 females were excluded due to history of orthodontic treatment. Furthermore, unsatisfactory image quality led to the exclusion of 9 frontal photos (5 females and 4 males) and 10 lateral photos (9 females and 1 males). As a result, there were respectively 266 (149 females and 117 males) and 265 (145 females and 120 males) participants from whom frontal and lateral images for all three phases of this study were available. Among them, 256 participants (140 females and 116 males) had complete photographic records from both frontal and lateral views.

The study protocol was approved by the Institutional Review Board of the University of Hong Kong/Hospital Authority Hong Kong West Cluster (IRB reference number: UW 13–584).

### Photographic set-up and record-taking

The photographic setup consisted of a tripod that held a digital single-lens reflex camera (Canon EOS 500D). The tripod allowed for stability during photographing and adjustment to its height enabled the optical axis of the lens to be kept at the same level as the participant’s eyes. A 100 mm focal lens was used to avoid major facial deformations. A primary flash (Canon Speedlite 430EX Flash unit) was set 45 cm to the right of the camera to the participant to avoid the “red-eye effect” on the photographs. A blue backdrop stood 3 m in frontal of the camera lens. A secondary flash (NiceFoto GY-120 studio lighting flash) was set at the left side of the backdrop at an anteroposterior distance of 1 m to eliminate shadows in the backdrop. The shutter speed was set at 1/125 second, the size of the diaphragm was adjusted to f/11, and the ISO speed was set at 100.

Each participant stood on a line 2 m in front of the camera lens. When the participant was faced with the camera, to their right hung a plumb line that indicated the true vertical (TV) direction. Next to the plumb line, a stainless steel ruler with 1 mm segments was freely dropped to allow for rescaling of the images to life-size. Approximately 1.1 m to the left of the participant stood a vertical mirror.

One frontal and one right-side profile image were taken for each participant. The photographs were taken while the participants assumed natural head position [31]. Participants were instructed to stand upright and look straight ahead into the camera lens while taking frontal images and into images of their own eyes in the mirror while taking lateral images. They tilted their heads forward and backward with a decreasing amplitude until the most neutral position was found [32]. The teeth were in centric occlusion and lips were gently closed. Eyeglasses were removed and no make-up was allowed. The forehead, ear, and neck were sufficiently exposed.

### Landmarks and measurements

**Fig 1:**
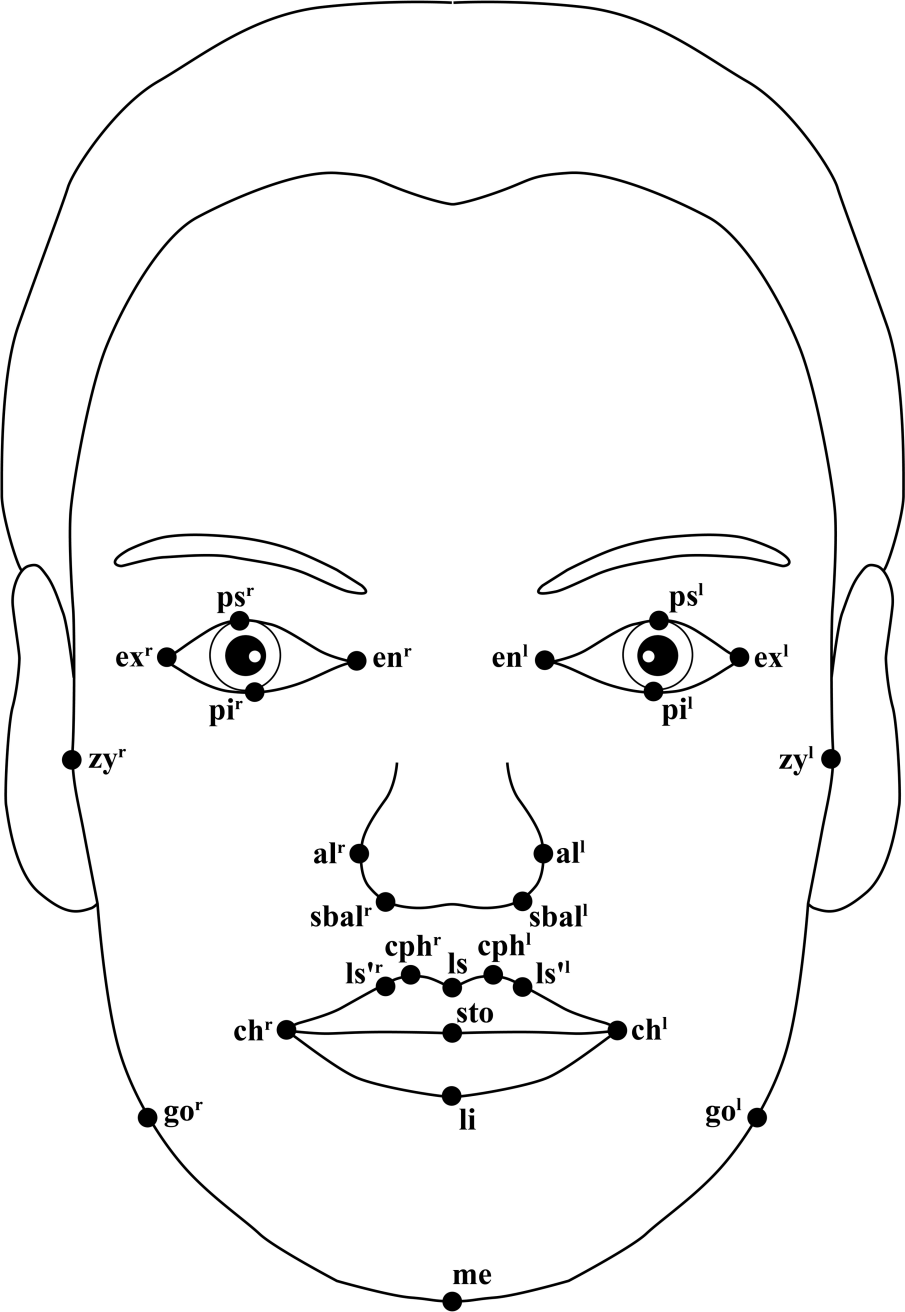
Schematic illustration of the anthropometric landmarks used on frontal facial photographs. ^l^The left of the bilaterally homologous landmarks; ^r^the right of the bilaterally homologous landmarks.

**Fig 2:**
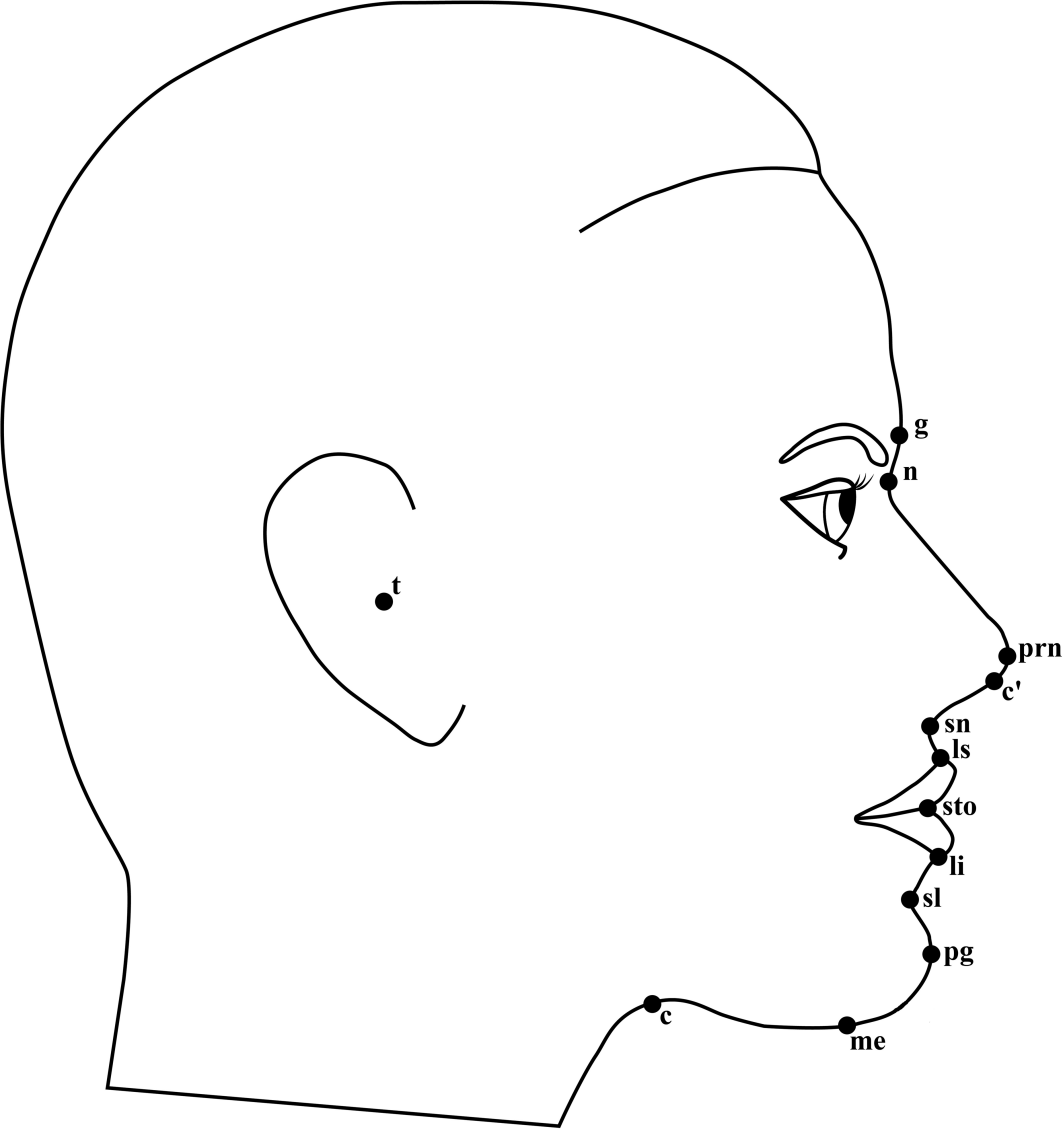
Schematic illustration of the anthropometric landmarks used on lateral facial photographs.

The photographic records were transferred to a personal computer running 64-bit version of Windows 7 and were then imported into tpsDig2, version 2.21 [33]. Using the software, two points separated by a distance of 50 mm along the vertically dropped ruler were marked on the image. Then, “50 mm” was manually input into the software, which calculated scale factor of the image. The landmarks used in this study were digitized according to the definitions by Farkas [34] and Naini [31] (S1 Table) and were illustrated in Figs 1 and 2. The order in which the landmarks were digitized was the same for each image. Upon completion of digitization for each image, two-dimensional Cartesian coordinates of the landmarks and the scale factor were stored. The landmarks were digitized by one trained investigator (YFW) and were checked for accuracy by another (HMW).

From the Cartesian coordinates, various linear and angular measurements, profile inclinations, and proportion indices were derived (S2 Table). Linear measurements derived from the Cartesian coordinates were scaled to life-size by multiplying the corresponding scale factor. All linear measurements were expressed in millimeters and angular measurements were expressed by angles in degrees. Profile inclinations were expressed in degrees of deviation from the TV. Positive inclination indicated that the lower landmark was prior to the vertical line dropped from the upper landmark, and vice versa. An inclination of 0 suggested that the upper and lower landmarks were perfectly vertically aligned. Percentage values were reported for proportion indices.

With the intention to provide a comprehensive description of facial growth, parameters for the facial, orbital, nasal, and orolabial region were selected from the standard anthropometric measurements described by Farkas [34, 35] and Powell [36]. Proportion indices involving parameters from more than one facial region were calculated and listed under the category “Cross-regional” in S2 Table.

### Examiner reliability

For assessment of examiner reliability, frontal and lateral photos of 50 randomly selected participants (25 females and 25 males) were digitized four times by one investigator (YFW), twice before and twice after digitizing the whole series of photographs. The replicate assessments were separated by an interval of at least two weeks.

Mean values for measurements from the first two replications, together with mean values from the last two replications, were obtained and used to calculate method error (ME) and method of moments (MME).

ME was calculated using the Dahlberg’s formula [37]:

$$ME=\sqrt{\frac{\sum_{i=1}^{n}d_{i}^{2}}{2n}}$$

where *d*_*i*_ is the difference between mean value for the first two and last two replications for the *i*^*th*^ participant, and *n* is the total sample size for replication assessment, which is 50 in this study.

MME was calculated using the following formula [38]:

$$MME=\sqrt{\frac{\sum_{i=1}^{n}{\left(d_{i}-\bar{d}\right)}^{2}}{2(n-1)}}$$

where *d*_*i*_ is the difference between mean value for the first two and last two replications for the *i*^*th*^ participant, $\bar{d}=\frac{\sum_{i=1}^{n}d_{i}}{n}$, and *n* is the total sample size for replication assessment, which is 50 in this study.

To determine the degree of agreement among the four replications, intraclass correlation coefficient (ICC) was calculated for each facial measurement. Two-way random model for single measurement, corresponding to the ICC(2,1) type of Shrout and Fleiss’ classification of 6 types of ICC [39], was used to derive the ICC values.

### Statistical analyses

To investigate changes of facial measurements from age 12 through 15 to 18 years, normality of data from each phase was checked using the Shapiro-Wilk test. When data were normally distributed, one-way repeated measures ANOVA was performed. Greenhouse-Geisser correction was employed if Mauchly’s test indicated violation of sphericity. Post-hoc pairwise comparisons were performed with Bonferroni corrections. When data were not normally distributed, Friedman test supplemented by Nemenyi post-hoc test was performed. Partial *ω*^2^, which reflected the proportion of variability in facial measurements explained by age, was used as a measure of effect size and was calculated using the formula $\omega^{2}=\frac{\left(k-1)(F-1\right)}{(k-1)(F-1)+nk}$, where *k* is the number of levels of the within-subjects factor, which is 3 in this study, *F* is the value of the F-statistic, and *n* is the sample size for the facial measurement [29]. Effect size for Friedman test was not calculated due to the lack of appropriate formula and it has also been suggested that effect size for Friedman test is not useful [40].

To statistically determine gender differences in the amount of growth changes of facial measurements from 12 to 15 years, from 15 to 18 years, and from 12 to 18 years, growth data from both genders were checked for normality using the Shapiro-Wilk test. When data were normally distributed, independent two-sample t-test was used if Levene’s test showed equal variances between genders while Welch’s t-test was used when there were unequal variances. When data were not normally distributed, median difference between genders was calculated and Mann-Whitney U test was used. To quantify the magnitude of difference between genders [41], point-biserial correlation (*r*) was used as a measure for effect size [42]. For independent two-sample t-test and Welch’s t-test, effect size was calculated using the formula $r=\sqrt{\frac{t^{2}}{t^{2}+df}}$, where *t* is the test statistic from the corresponding t-test and *df* is the degree of freedom [43]. For Mann-Whitney U test, effect size was calculated using $r=\frac{Z}{\sqrt{n}}$, where *Z* is the Z-score for the U-value and *n* is the sample size for the facial measurement [44].

The cut-offs for small, medium, and large effect were 0.06 and 0.15 for partial *ω*^2^ [29] and were 0.30 and 0.50 for point-biserial correlation (*r*) [45]. The level of statistical significance was set at 0.05. Sample size calculation was performed using the G*Power software (version 3.1.9.2; University of Düsseldorf, Düsseldorf, Germany). All statistical analyses were performed in R version 3.2.2 [46].

## Results

### Examiner reliability

Results of intra-examiner reliability are shown in S3 Table. ME for all linear measurements were below 0.31 mm. The only two angular measurements with ME larger than 2.00° were the nasolabial angle (2.19°) and labiomental angle (2.43°). There were six regional and two cross-regional facial indices whose ME were higher than 1.00%. Among these, six were related to lips, two were related to chin, and two were related to nose. Values for MME were generally smaller than values for ME, but both showed similar patterns of reliability. Except for the mandible height-lower third face depth index (ICC: 0.79) and nasal tip angle (ICC: 0.84), ICC was greater than 0.85 all other facial measurements, suggesting that the intra-examiner agreement was excellent, according to Cicchetti et al [47]’s guideline of cut-offs for ICC.

### Longitudinal changes of facial parameters

Findings on statistical significance of changes in facial parameters from 12 through 15 to 18 years of age, together with pairwise comparisons between age levels, are shown in Table 1 for females and Table 2 for males. All original data are available from S4–S9 Tables.

**Table 1:** Changes of photogrammetric measurements from 12 through 15 to 18 years of age for females.

Measurement		*n*	Overall significance						Pairwise comparisons											
									12 vs 15				15 vs 18				12 vs 18			
			*F*	*df*_*M*_	*df*_*E*_	*p*-value		*ES*	$\Delta \bar{x}$	*SE*	*p*-value		$\Delta \bar{x}$	*SE*	*p*-value		$\Delta \bar{x}$	*SE*	*p*-value	
Face																				
	Width of the face	149	19.06	1.55	229.24	<0.001	***	0.07	1.8	0.3	<0.001	***	0.3	0.4	1.000		2.0	0.4	<0.001	***
	Width of the mandible	149	12.16	2.00	296.00	<0.001	***	0.05	2.0	0.5	<0.001	***	0.5	0.5	0.901		2.5	0.6	<0.001	***
	Height of the face	145	59.43^†^	..	..	<0.001	***	..	1.9	..	0.504		3.1	..	<0.001	***	5.0	..	<0.001	***
	Height of the upper face	145	80.33^†^	..	..	<0.001	***	..	0.9	..	0.885		1.9	..	<0.001	***	2.9	..	<0.001	***
	Height of the lower face	145	52.73^†^	..	..	<0.001	***	..	1.3	..	0.725		1.6	..	<0.001	***	2.9	..	<0.001	***
	Height of the mandible	145	25.75^†^	..	..	<0.001	***	..	1.2	..	0.204		0.3	..	0.003	**	1.6	..	<0.001	***
	Height of the chin	145	33.89^†^	..	..	<0.001	***	..	0.9	..	0.004	**	0.6	..	0.022	*	1.4	..	<0.001	***
	Height of the lower profile	145	5.34^†^	..	..	0.069		..	1.4	..	0.336		0.3	..	0.652		1.7	..	0.057	
	Height of the midface	145	11.44	1.48	212.73	<0.001	***	0.05	1.0	0.4	0.076		0.8	0.2	0.003	**	1.8	0.4	<0.001	***
	Lower half of the craniofacial height (left)	149	59.37^†^	..	..	<0.001	***	..	1.4	..	<0.001	***	2.2	..	<0.001	***	3.7	..	<0.001	***
	Lower half of the craniofacial height (right)	149	34.72	1.70	251.34	<0.001	***	0.13	0.5	0.2	0.085		1.7	0.3	<0.001	***	2.2	0.3	<0.001	***
	Mentocervical angle	145	13.24	1.85	266.68	<0.001	***	0.05	-0.2	0.5	1.000		2.0	0.4	<0.001	***	1.8	0.5	<0.001	***
	Angle of facial convexity	145	4.35	1.62	233.89	0.020	*	0.02	0.3	0.2	0.411		-0.6	0.2	<0.001	***	-0.3	0.2	0.726	
	Angle of total facial convexity	145	0.98	1.57	225.45	0.359		0.00	-0.2	0.2	1.000		-0.1	0.1	1.000		-0.2	0.2	0.683	
	Angle of the medium facial third	145	29.08	1.82	261.63	<0.001	***	0.11	0.0	0.2	1.000		-1.1	0.1	<0.001	***	-1.0	0.2	<0.001	***
	Angle of the inferior facial third	145	56.02	1.73	249.81	<0.001	***	0.20	0.1	0.2	1.000		-1.7	0.1	<0.001	***	-1.6	0.2	<0.001	***
	Inclination of general profile line	145	32.00	1.85	266.25	<0.001	***	0.12	2.6	0.3	<0.001	***	-2.0	0.3	<0.001	***	0.6	0.4	0.340	
	Inclination of upper face profile line	145	27.07	1.88	270.50	<0.001	***	0.11	2.5	0.3	<0.001	***	-1.7	0.3	<0.001	***	0.8	0.4	0.136	
	Inclination of lower face profile line	145	31.89	1.77	254.20	<0.001	***	0.12	2.8	0.4	<0.001	***	-2.3	0.3	<0.001	***	0.5	0.4	0.738	
	Inclination of lower third face line	145	24.51	1.82	261.85	<0.001	***	0.10	3.9	0.6	<0.001	***	-3.1	0.5	<0.001	***	0.8	0.7	0.710	
	Inclination of the chin	145	36.94^†^	..	..	<0.001	***	..	2.3	..	<0.001	***	-2.7	..	<0.001	***	-0.4	..	0.689	
	Facial index	140	34.90^†^	..	..	<0.001	***	..	-0.2	..	0.952		1.7	..	<0.001	***	1.6	..	<0.001	***
	Mandible-face width index	149	2.75	2.00	296.00	0.065		0.01	0.5	0.3	0.378		0.2	0.3	1.000		0.7	0.3	0.088	
	Upper face index	140	18.09	1.46	202.53	<0.001	***	0.08	-0.1	0.3	1.000		1.5	0.2	<0.001	***	1.3	0.3	<0.001	***
	Mandible width-face height index	140	7.90^†^	..	..	0.019	*	..	1.3	..	0.294		-1.7	..	0.014	*	-0.4	..	0.387	
	Mandibular index	140	3.66^†^	..	..	0.161		..	0.5	..	0.605		-0.1	..	0.135		0.4	..	0.605	
	Upper face-face height index	145	5.70	1.88	270.93	0.005	**	0.02	-0.2	0.1	0.510		0.4	0.1	<0.001	***	0.2	0.1	0.189	
	Lower face-face height index	145	7.84	1.88	271.23	<0.001	***	0.03	0.3	0.1	0.028	*	0.1	0.1	0.621		0.4	0.1	0.001	**
	Chin-face height index	145	14.95^†^	..	..	<0.001	***	..	0.7	..	0.002	**	0.0	..	0.992		0.7	..	0.003	**
	Chin-mandible height index	145	21.94^†^	..	..	<0.001	***	..	1.5	..	0.002	**	0.4	..	0.578		1.9	..	<0.001	***
	Chin index	145	9.41^†^	..	..	0.009	**	..	2.0	..	0.954		-1.0	..	0.015	*	1.0	..	0.034	*
	Mandibulo-face height index	145	5.70	1.88	270.93	0.005	**	0.02	0.2	0.1	0.510		-0.4	0.1	<0.001	***	-0.2	0.1	0.189	
	Mandibulo-upper face height index	145	5.96	1.86	268.41	0.004	**	0.02	0.5	0.4	0.461		-1.1	0.3	<0.001	***	-0.6	0.3	0.177	
	Mandibulo-lower face height index	145	17.49	1.83	262.88	<0.001	***	0.07	0.0	0.2	1.000		-0.9	0.1	<0.001	***	-0.9	0.2	<0.001	***
	Mandible width-lower third face depth index	140	10.53	1.76	244.44	<0.001	***	0.04	2.6	0.7	<0.001	***	-2.4	0.5	<0.001	***	0.2	0.7	1.000	
	Upper face height-upper third face depth index	145	18.41	1.80	258.84	<0.001	***	0.07	2.0	0.5	<0.001	***	0.4	0.3	0.823		2.4	0.4	<0.001	***
	Mandible height-lower third face depth index	145	27.41^†^	..	..	<0.001	***	..	0.6	..	<0.001	***	-0.3	..	0.049	*	0.3	..	0.011	*
	Upper-middle third face depth index	145	6.57	1.38	198.37	0.005	**	0.02	-0.4	0.2	0.255		-0.3	0.1	0.011	*	-0.7	0.2	0.010	*
	Middle-lower third face depth index	145	1.11	1.83	263.06	0.327		0.00	-0.3	0.3	0.684		0.0	0.2	1.000		-0.3	0.3	0.698	
	Upper cheek-upper third face depth index	145	23.43^†^	..	..	<0.001	***	..	-0.8	..	<0.001	***	-0.2	..	0.725		-0.9	..	<0.001	***
Orbits																				
	Intercanthal width	149	31.40	1.56	230.45	<0.001	***	0.12	0.5	0.1	<0.001	***	0.2	0.1	0.224		0.6	0.1	<0.001	***
	Biocular width	149	22.20	1.33	197.04	<0.001	***	0.09	0.5	0.1	<0.001	***	0.7	0.2	0.005	**	1.2	0.2	<0.001	***
	Length of the eye fissure (left)	149	18.87	1.91	283.19	<0.001	***	0.07	0.3	0.1	0.001	**	0.2	0.1	0.012	*	0.5	0.1	<0.001	***
	Length of the eye fissure (right)	149	13.50	1.87	276.55	<0.001	***	0.05	0.1	0.1	0.135		0.3	0.1	0.007	**	0.4	0.1	<0.001	***
	Height of the eye fissure (left)	149	59.90	2.00	296.00	<0.001	***	0.21	0.4	0.1	<0.001	***	0.5	0.1	<0.001	***	1.0	0.1	<0.001	***
	Height of the eye fissure (right)	149	70.26	2.00	296.00	<0.001	***	0.24	0.5	0.1	<0.001	***	0.5	0.1	<0.001	***	1.0	0.1	<0.001	***
	Intercanthal index	149	11.25	1.79	264.25	<0.001	***	0.04	0.3	0.1	<0.001	***	-0.1	0.1	0.014	*	0.2	0.1	0.081	
	Orbital width index	149	0.67	1.73	256.12	0.491		0.00	-0.2	0.3	1.000		0.3	0.2	0.457		0.1	0.3	1.000	
	Eye fissure index	149	37.91	2.00	296.00	<0.001	***	0.14	1.3	0.3	0.001	**	1.8	0.3	<0.001	***	3.0	0.4	<0.001	***
Nose																				
	Width of the nose	149	16.16	1.87	276.58	<0.001	***	0.06	0.1	0.1	1.000		0.5	0.1	<0.001	***	0.6	0.1	<0.001	***
	Height of the nose	145	13.48	1.34	192.49	<0.001	***	0.05	0.3	0.3	1.000		1.1	0.1	<0.001	***	1.3	0.3	<0.001	***
	Length of the nasal bridge	145	1.87	1.40	201.26	0.169		0.00	0.2	0.3	1.000		0.3	0.2	0.136		0.5	0.3	0.338	
	Nasal protrusion	145	25.61^†^	..	..	<0.001	***	..	-0.1	..	0.652		0.3	..	<0.001	***	0.2	..	<0.001	***
	Nasofrontal angle	145	65.56^†^	..	..	<0.001	***	..	-0.1	..	0.099		-1.5	..	<0.001	***	-1.7	..	<0.001	***
	Nasal tip angle	145	7.30^†^	..	..	0.026	*	..	-0.1	..	0.367		0.2	..	0.367		0.1	..	0.019	*
	Nasolabial angle	145	5.31	1.82	262.78	0.007	**	0.02	1.9	0.7	0.039	*	0.1	0.6	1.000		1.9	0.7	0.020	*
	Nasofacial angle	145	36.35	1.73	249.65	<0.001	***	0.14	-0.1	0.1	1.000		1.0	0.1	<0.001	***	0.9	0.1	<0.001	***
	Nasomental angle	145	25.47	1.60	230.57	<0.001	***	0.10	0.1	0.2	1.000		-1.1	0.1	<0.001	***	-1.0	0.2	<0.001	***
	Inclination of nasal bridge	145	34.96	1.88	270.37	<0.001	***	0.14	2.4	0.3	<0.001	***	-0.9	0.3	0.002	**	1.5	0.3	<0.001	***
	Nasal index	140	3.93	1.45	201.10	0.034	*	0.01	-0.5	0.5	0.900		-0.7	0.3	0.036	*	-1.2	0.5	0.041	*
	Nostril-nose width index	149	22.02	1.91	282.79	<0.001	***	0.09	-0.5	0.3	0.324		2.3	0.4	<0.001	***	1.8	0.4	<0.001	***
	Nostril width-nose height index	140	25.73^†^	..	..	<0.001	***	..	-0.1	..	0.998		1.2	..	<0.001	***	1.1	..	<0.001	***
	Nasal tip protrusion-width index	140	15.03	1.52	210.69	<0.001	***	0.06	-0.9	0.3	0.008	**	-0.5	0.2	0.008	**	-1.5	0.3	<0.001	***
	Nasal tip protrusion-nostril floor width index	140	25.64	1.82	253.57	<0.001	***	0.11	-1.0	0.6	0.304		-3.4	0.6	<0.001	***	-4.4	0.7	<0.001	***
	Nasal tip protrusion-nose height index	145	0.96	1.69	243.20	0.372		0.00	-0.2	0.1	0.806		0.0	0.1	1.000		-0.2	0.2	0.844	
	Nasal bridge index	145	34.43	1.81	260.03	<0.001	***	0.13	-0.1	0.2	1.000		-1.2	0.2	<0.001	***	-1.3	0.2	<0.001	***
Lips and mouth																				
	Width of the philtrum	149	13.25	2.00	296.00	<0.001	***	0.05	0.0	0.1	1.000		0.5	0.1	<0.001	***	0.5	0.1	<0.001	***
	Width of the mouth	149	19.26	2.00	296.00	<0.001	***	0.08	1.4	0.3	<0.001	***	0.2	0.3	1.000		1.6	0.3	<0.001	***
	Height of the upper lip	145	99.49^†^	..	..	<0.001	***	..	0.4	..	0.306		1.1	..	<0.001	***	1.5	..	<0.001	***
	Height of the cutaneous upper lip	145	34.81^†^	..	..	<0.001	***	..	0.2	..	0.992		0.7	..	<0.001	***	0.9	..	<0.001	***
	Vermilion height of the upper lip	145	26.36^†^	..	..	<0.001	***	..	0.3	..	0.434		0.4	..	<0.001	***	0.8	..	<0.001	***
	Vermilion height of the lower lip	145	12.68^†^	..	..	0.002	**	..	-0.4	..	0.998		0.5	..	0.006	**	0.1	..	0.005	**
	Height of the cutaneous lower lip	145	0.96	2.00	288.00	0.384		0.00	0.1	0.1	1.000		0.1	0.1	1.000		0.2	0.1	0.610	
	Height of the lower lip	145	18.17^†^	..	..	<0.001	***	..	0.1	..	0.652		0.5	..	0.004	**	0.6	..	<0.001	***
	Labiomental angle	145	0.59	2.00	288.00	0.556		0.00	0.9	0.9	0.962		-0.5	0.8	1.000		0.4	0.8	1.000	
	Inclination of upper lip	145	2.56	1.88	271.44	0.083		0.01	0.4	0.7	1.000		-1.4	0.6	0.044	*	-1.0	0.6	0.373	
	Inclination of lower lip	145	13.66	2.00	288.00	<0.001	***	0.05	3.7	0.8	<0.001	***	-3.1	0.7	<0.001	***	0.5	0.8	1.000	
	Upper lip height-mouth width index	140	8.91	1.72	238.39	<0.001	***	0.04	-0.7	0.6	0.729		2.0	0.4	<0.001	***	1.4	0.5	0.022	*
	Mouth width contour index	149	3.76	1.80	265.66	0.029	*	0.01	0.1	0.1	0.2		0.0	0.0	1.000		0.1	0.1	0.054	
	Philtrum-mouth width index	149	8.21	2.00	296.00	<0.001	***	0.03	-0.7	0.3	0.021	*	1.1	0.3	<0.001	***	0.3	0.3	0.689	
	Medial-lateral cutaneous upper lip height index	140	9.56	1.83	254.99	<0.001	***	0.04	-1.7	1.1	0.407		4.3	0.9	<0.001	***	2.6	0.9	0.021	*
	Vermilion-total upper lip height index	145	0.22	1.72	247.82	0.770		0.00	0.4	0.6	1.000		-0.2	0.5	1.000		0.1	0.5	1.000	
	Vermilion height index	145	3.85^†^	..	..	0.146		..	1.0	..	0.761		0.5	..	0.434		1.5	..	0.128	
	Upper vermilion contour index	149	139.45^†^	..	..	<0.001	***	..	4.3	..	<0.001	***	1.5	..	0.003	**	5.8	..	<0.001	***
	Lower vermilion contour index	149	378.98	2.00	296.00	<0.001	***	0.63	3.1	0.2	<0.001	***	3.3	0.2	<0.001	***	6.4	0.2	<0.001	***
	Lower-upper lip height index	145	15.82^†^	..	..	<0.001	***	..	-0.1	..	0.954		-1.8	..	0.003	**	-2.0	..	<0.001	***
	Cutaneous lower-upper lip height index	145	3.74^†^	..	..	0.154		..	1.4	..	0.998		-4.3	..	0.227		-2.9	..	0.204	
	Vermilion-total lower lip height index	145	0.50	2.00	288.00	0.606		0.00	-0.1	0.7	1.000		0.6	0.6	1.000		0.5	0.7	1.000	
	Vermilion arch index	149	8.63^†^	..	..	0.013	*	..	0.9	..	0.053		-1.8	..	0.018	*	-0.9	..	0.913	
Cross-regional																				
	Upper face height-biocular width index	140	18.18	1.50	209.12	<0.001	***	0.08	0.3	0.5	1.000		1.9	0.3	<0.001	***	2.3	0.5	<0.001	***
	Biocular-face width index	149	9.99	1.67	247.49	<0.001	***	0.04	-0.4	0.1	<0.001	***	0.4	0.1	<0.001	***	-0.1	0.1	1.000	
	Intercanthal width-upper face height index	140	19.06	1.48	205.42	<0.001	***	0.08	0.0	0.3	1.000		-1.4	0.2	<0.001	***	-1.4	0.3	<0.001	***
	Intercanthal-nasal width index	149	10.08	1.87	276.38	<0.001	***	0.04	1.1	0.3	<0.001	***	-0.9	0.2	<0.001	***	0.2	0.3	1.000	
	Intercanthal-mouth width index	149	7.55	1.84	271.68	<0.001	***	0.03	-1.7	0.6	0.012	*	0.0	0.4	1.000		-1.7	0.5	0.002	**
	Nose-face width index	149	12.30	1.89	279.85	<0.001	***	0.05	-0.3	0.1	<0.001	***	0.3	0.1	<0.001	***	0.0	0.1	1.000	
	Nose-mouth width index	149	22.83^†^	..	..	<0.001	***	..	-2.9	..	<0.001	***	0.8	..	0.261		-2.1	..	0.005	**
	Nose height-face width index	140	7.66	1.45	201.31	0.002	**	0.03	-0.2	0.2	0.867		0.7	0.1	<0.001	***	0.5	0.2	0.064	
	Nose-face height index	145	7.84	1.88	271.23	<0.001	***	0.03	-0.3	0.1	0.028	*	-0.1	0.1	0.621		-0.4	0.1	0.001	**
	Nose-upper face height index	145	27.66	1.82	261.95	<0.001	***	0.11	-0.2	0.1	0.277		-0.7	0.1	<0.001	***	-0.9	0.1	<0.001	***
	Nose-lower face height index	145	7.35	1.88	271.05	0.001	**	0.03	-0.9	0.3	0.036	*	-0.4	0.3	0.619		-1.2	0.4	0.002	**
	Nasal tip protrusion-upper lip height index	145	20.92	1.79	257.06	<0.001	***	0.08	-1.2	0.6	0.136		-2.3	0.4	<0.001	***	-3.5	0.6	<0.001	***
	Mouth-face width index	149	7.80	1.89	279.46	<0.001	***	0.03	0.6	0.2	0.016	*	0.1	0.2	1.000		0.7	0.2	<0.001	***
	Upper lip-upper face height index	145	27.66	1.82	261.95	<0.001	***	0.11	0.2	0.1	0.277		0.7	0.1	<0.001	***	0.9	0.1	<0.001	***
	Upper lip-mandible height index	145	17.24	1.85	265.70	<0.001	***	0.07	0.0	0.4	1.000		2.0	0.3	<0.001	***	2.0	0.4	<0.001	***
	Upper lip-nose height index	145	41.34^†^	..	..	<0.001	***	..	1.0	..	0.857		0.8	..	<0.001	***	1.8	..	<0.001	***
	Lower lip-face height index	145	1.75^†^	..	..	0.417		..	-0.4	..	0.468		0.2	..	0.998		-0.2	..	0.504	
	Lower lip-mandible height index	145	8.37^†^	..	..	0.015	*	..	-0.6	..	0.204		0.6	..	0.011	*	0.1	..	0.468	
	Lower lip-chin height index	145	5.02^†^	..	..	0.081		..	-2.8	..	0.066		0.6	..	0.615		-2.2	..	0.400	

***p < 0.001.

**p < 0.01.

*p < 0.05.

*n* = sample size; *F* = *F*-value; *df*_*M*_ = model degree of freedom; *df*_*E*_ = error degree of freedom; *ES* = effect size; $\Delta \bar{x}$ = mean difference (measurement at the younger age was subtracted from the same measurement at the older age); *SE* = standard error.

†: due to non-normality of the data, Friedman test statistic was used to derive the *F*-value. Pairwise median differences were reported and statistically tested using Nemenyi post-hoc test.

**Table 2:** Changes of photogrammetric measurements from 12 through 15 to 18 years of age for males.

Measurement		*n*	Overall significance						Pairwise comparisons											
									12 vs 15				15 vs 18				12 vs 18			
			*F*	*df*_*M*_	*df*_*E*_	*p*-value		*ES*	$\Delta \bar{x}$	*SE*	*p*-value		$\Delta \bar{x}$	*SE*	*p*-value		$\Delta \bar{x}$	*SE*	*p*-value	
Face																				
	Width of the face	117	0.35	1.53	177.76	0.648		0.00	0.2	0.3	1.000		0.2	0.4	1.000		0.4	0.5	1.000	
	Width of the mandible	117	6.89^†^	..	..	0.032	*	..	2.7	..	0.091		-1.2	..	0.943		1.5	..	0.041	*
	Height of the face	120	74.47^†^	..	..	<0.001	***	..	3.6	..	0.038	*	2.7	..	<0.001	***	6.3	..	<0.001	***
	Height of the upper face	120	42.75	1.29	153.60	<0.001	***	0.19	0.8	0.4	0.216		2.6	0.2	<0.001	***	3.3	0.5	<0.001	***
	Height of the lower face	120	40.44	1.38	163.86	<0.001	***	0.18	1.9	0.5	<0.001	***	2.0	0.2	<0.001	***	3.9	0.5	<0.001	***
	Height of the mandible	120	20.66	1.43	169.82	<0.001	***	0.10	1.3	0.4	0.004	**	0.9	0.2	<0.001	***	2.1	0.4	<0.001	***
	Height of the chin	120	27.35^†^	..	..	<0.001	***	..	1.6	..	0.018	*	0.5	..	0.032	*	2.1	..	<0.001	***
	Height of the lower profile	120	8.22	1.38	164.81	0.002	**	0.04	0.9	0.6	0.401		1.1	0.3	<0.001	***	2.0	0.6	0.002	**
	Height of the midface	120	6.59	1.49	177.82	0.004	**	0.03	0.1	0.4	1.000		1.1	0.2	<0.001	***	1.2	0.4	0.016	*
	Lower half of the craniofacial height (left)	117	86.92	1.62	187.41	<0.001	***	0.33	2.9	0.3	<0.001	***	1.7	0.4	<0.001	***	4.7	0.4	<0.001	***
	Lower half of the craniofacial height (right)	117	54.44^†^	..	..	<0.001	***	..	2.0	..	<0.001	***	1.9	..	0.098		4.0	..	<0.001	***
	Mentocervical angle	120	16.25^†^	..	..	<0.001	***	..	-2.7	..	<0.001	***	1.0	..	0.010	*	-1.7	..	0.597	
	Angle of facial convexity	120	0.53	1.53	181.52	0.541		0.00	-0.2	0.3	1.000		0.2	0.2	0.478		0.0	0.3	1.000	
	Angle of total facial convexity	120	12.01	1.36	162.12	<0.001	***	0.06	-1.1	0.3	<0.001	***	0.3	0.1	0.103		-0.8	0.3	0.013	*
	Angle of the medium facial third	120	20.46	1.73	205.45	<0.001	***	0.10	-0.1	0.2	1.000		-0.9	0.1	<0.001	***	-1.0	0.2	<0.001	***
	Angle of the inferior facial third	120	7.32	1.73	205.30	0.002	**	0.03	0.6	0.2	0.004	**	-0.1	0.1	1.000		0.6	0.2	0.021	*
	Inclination of general profile line	120	4.49	2.00	238.00	0.012	*	0.02	1.1	0.4	0.017	*	-0.1	0.4	1.000		1.0	0.4	0.078	
	Inclination of upper face profile line	120	4.55	2.00	238.00	0.011	*	0.02	1.2	0.4	0.013	*	-0.2	0.4	1.000		1.0	0.5	0.113	
	Inclination of lower face profile line	120	4.34	1.86	221.91	0.016	*	0.02	1.0	0.4	0.047	*	0.1	0.4	1.000		1.1	0.5	0.053	
	Inclination of lower third face line	120	1.77	1.68	200.34	0.179		0.00	1.2	0.7	0.275		-0.6	0.5	0.589		0.6	0.7	1.000	
	Inclination of the chin	120	0.12^†^	..	..	0.943		..	-0.2	..	0.964		-1.1	..	0.998		-1.3	..	0.944	
	Facial index	116	57.95^†^	..	..	<0.001	***	..	1.8	..	0.066		1.8	..	<0.001	***	3.6	..	<0.001	***
	Mandible-face width index	117	9.07	2.00	232.00	<0.001	***	0.04	1.2	0.3	<0.001	***	0.1	0.3	1.000		1.2	0.3	0.002	**
	Upper face index	116	33.94	1.43	164.55	<0.001	***	0.16	0.6	0.3	0.259		1.6	0.2	<0.001	***	2.2	0.3	<0.001	***
	Mandible width-face height index	116	10.40	1.51	173.95	<0.001	***	0.05	-0.5	0.8	1.000		-2.4	0.4	<0.001	***	-2.9	0.7	<0.001	***
	Mandibular index	116	6.55	1.53	176.30	0.004	**	0.03	0.6	0.4	0.423		0.6	0.2	0.018	*	1.2	0.4	0.004	**
	Upper face-face height index	120	5.83	1.77	210.60	0.005	**	0.03	-0.5	0.1	0.005	**	0.3	0.1	0.012	*	-0.1	0.2	1.000	
	Lower face-face height index	120	18.29	1.72	205.07	<0.001	***	0.09	0.6	0.1	<0.001	***	0.0	0.1	1.000		0.7	0.1	<0.001	***
	Chin-face height index	120	5.73	1.71	203.45	0.006	**	0.03	0.5	0.2	0.020	*	0.0	0.1	1.000		0.5	0.2	0.033	*
	Chin-mandible height index	120	5.99	1.64	195.27	0.005	**	0.03	0.8	0.4	0.173		0.5	0.3	0.235		1.2	0.4	0.007	**
	Chin index	120	7.93^†^	..	..	0.019	*	..	-0.6	..	0.268		-6.3	..	0.419		-6.9	..	0.014	*
	Mandibulo-face height index	120	5.83	1.77	210.60	0.005	**	0.03	0.5	0.1	0.005	**	-0.3	0.1	0.012	*	0.1	0.2	1.000	
	Mandibulo-upper face height index	120	5.72	1.79	212.64	0.005	**	0.03	1.2	0.4	0.006	**	-0.9	0.3	0.011	*	0.3	0.4	1.000	
	Mandibulo-lower face height index	120	6.98	1.73	205.64	0.002	**	0.03	0.1	0.2	1.000		-0.6	0.1	<0.001	***	-0.5	0.2	0.028	*
	Mandible width-lower third face depth index	116	7.57^†^	..	..	0.023	*	..	1.3	..	0.670		-3.1	..	0.019	*	-1.8	..	0.157	
	Upper face height-upper third face depth index	120	9.08	1.72	204.46	<0.001	***	0.04	1.6	0.5	0.005	**	0.1	0.4	1.000		1.7	0.5	0.002	**
	Mandible height-lower third face depth index	120	15.32^†^	..	..	<0.001	***	..	1.0	..	0.002	**	0.1	..	0.998		1.1	..	0.002	**
	Upper-middle third face depth index	120	23.91	1.36	161.35	<0.001	***	0.11	-1.2	0.3	<0.001	***	-0.3	0.1	0.114		-1.4	0.3	<0.001	***
	Middle-lower third face depth index	120	18.12^†^	..	..	<0.001	***	..	-0.9	..	0.964		1.2	..	<0.001	***	0.4	..	0.001	**
	Upper cheek-upper third face depth index	120	39.69	1.55	184.64	<0.001	***	0.18	-1.7	0.3	<0.001	***	-0.4	0.2	0.080		-2.1	0.3	<0.001	***
Orbits																				
	Intercanthal width	117	39.21	1.61	186.39	<0.001	***	0.18	0.5	0.1	<0.001	***	0.5	0.1	<0.001	***	1.0	0.1	<0.001	***
	Biocular width	117	59.02	1.56	181.00	<0.001	***	0.25	0.5	0.2	0.005	**	1.9	0.2	<0.001	***	2.4	0.3	<0.001	***
	Length of the eye fissure (left)	117	66.82	1.83	212.86	<0.001	***	0.27	0.3	0.1	0.017	*	0.9	0.1	<0.001	***	1.2	0.1	<0.001	***
	Length of the eye fissure (right)	117	31.50^†^	..	..	<0.001	***	..	0.4	..	0.091		0.3	..	0.002	**	0.7	..	<0.001	***
	Height of the eye fissure (left)	117	49.97^†^	..	..	<0.001	***	..	0.4	..	0.024	*	0.6	..	<0.001	***	1.0	..	<0.001	***
	Height of the eye fissure (right)	117	48.62^†^	..	..	<0.001	***	..	0.3	..	0.259		0.7	..	<0.001	***	1.0	..	<0.001	***
	Intercanthal index	117	5.43	1.44	167.31	0.011	*	0.02	0.3	0.1	0.043	*	-0.3	0.1	<0.001	***	0.0	0.1	1.000	
	Orbital width index	117	11.33	1.50	173.73	<0.001	***	0.06	-0.1	0.4	1.000		1.4	0.2	<0.001	***	1.3	0.4	0.002	**
	Eye fissure index	117	10.44	1.75	203.36	<0.001	***	0.05	0.6	0.4	0.264		1.0	0.3	0.003	**	1.6	0.4	<0.001	***
Nose																				
	Width of the nose	117	4.23	1.74	202.01	0.020	*	0.02	0.1	0.1	1.000		0.3	0.1	0.068		0.5	0.2	0.056	
	Height of the nose	120	59.52^†^	..	..	<0.001	***	..	0.7	..	0.998		1.2	..	<0.001	***	1.9	..	<0.001	***
	Length of the nasal bridge	120	8.88	1.31	156.32	0.001	**	0.04	0.6	0.3	0.150		0.5	0.1	0.001	**	1.1	0.3	0.002	**
	Nasal protrusion	120	0.39	1.32	157.36	0.590		0.00	0.0	0.1	1.000		0.1	0.1	0.480		0.1	0.1	1.000	
	Nasofrontal angle	120	135.80^†^	..	..	<0.001	***	..	-3.7	..	<0.001	***	-2.3	..	<0.001	***	-6.0	..	<0.001	***
	Nasal tip angle	120	65.60	1.55	184.36	<0.001	***	0.26	-3.5	0.5	<0.001	***	-1.1	0.3	<0.001	***	-4.6	0.5	<0.001	***
	Nasolabial angle	120	0.54	1.60	190.31	0.542		0.00	0.6	0.7	1.000		-0.5	0.5	0.896		0.2	0.7	1.000	
	Nasofacial angle	120	59.20	1.52	181.10	<0.001	***	0.24	1.1	0.2	<0.001	***	0.4	0.1	<0.001	***	1.5	0.2	<0.001	***
	Nasomental angle	120	47.26	1.38	164.48	<0.001	***	0.20	-1.6	0.2	<0.001	***	-0.2	0.1	0.145		-1.9	0.3	<0.001	***
	Inclination of nasal bridge	120	28.60	2.00	238.00	<0.001	***	0.13	2.2	0.4	<0.001	***	0.3	0.3	1.000		2.5	0.4	<0.001	***
	Nasal index	116	15.02^†^	..	..	<0.001	***	..	-0.5	..	0.963		-0.9	..	0.004	**	-1.4	..	0.001	**
	Nostril-nose width index	117	42.60	2.00	232.00	<0.001	***	0.19	-1.9	0.3	<0.001	***	3.3	0.3	<0.001	***	1.4	0.4	<0.001	***
	Nostril width-nose height index	116	23.14	1.83	210.55	<0.001	***	0.11	-0.5	0.3	0.132		1.5	0.2	<0.001	***	1.0	0.2	<0.001	***
	Nasal tip protrusion-width index	116	2.18	1.54	177.24	0.128		0.01	-0.3	0.3	1.000		-0.3	0.2	0.303		-0.6	0.3	0.215	
	Nasal tip protrusion-nostril floor width index	116	43.10^†^	..	..	<0.001	***	..	1.8	..	0.003	**	-3.5	..	<0.001	***	-1.7	..	0.003	**
	Nasal tip protrusion-nose height index	120	14.52	1.56	185.80	<0.001	***	0.07	-0.1	0.2	1.000		-0.7	0.1	<0.001	***	-0.8	0.2	<0.001	***
	Nasal bridge index	120	18.80	1.83	217.70	<0.001	***	0.09	0.8	0.2	0.003	**	-1.4	0.2	<0.001	***	-0.6	0.3	0.073	
Lips and mouth																				
	Width of the philtrum	117	5.91	2.00	232.00	0.003	**	0.03	0.4	0.1	0.009	**	0.0	0.1	1.000		0.4	0.1	0.010	**
	Width of the mouth	117	1.64	1.90	220.79	0.197		0.00	0.3	0.3	0.810		0.2	0.3	1.000		0.6	0.3	0.317	
	Height of the upper lip	120	55.42	1.60	189.97	<0.001	***	0.23	0.6	0.2	0.004	**	1.1	0.1	<0.001	***	1.7	0.2	<0.001	***
	Height of the cutaneous upper lip	120	13.00	1.72	204.83	<0.001	***	0.06	0.0	0.2	1.000		0.7	0.1	<0.001	***	0.7	0.2	<0.001	***
	Vermilion height of the upper lip	120	23.83	1.80	213.73	<0.001	***	0.11	0.6	0.2	0.002	**	0.4	0.1	0.002	**	1.0	0.2	<0.001	***
	Vermilion height of the lower lip	120	7.72^†^	..	..	0.021	*	..	0.0	..	0.298		0.5	..	0.400		0.5	..	0.015	*
	Height of the cutaneous lower lip	120	2.05	1.68	199.72	0.139		0.01	0.3	0.2	0.539		0.1	0.1	1.000		0.4	0.2	0.254	
	Height of the lower lip	120	4.96	1.45	172.55	0.016	*	0.02	0.4	0.2	0.208		0.2	0.1	0.358		0.6	0.2	0.023	*
	Labiomental angle	120	2.48	1.75	207.92	0.094		0.01	2.0	1.0	0.162		-0.3	0.8	1.000		1.7	1.1	0.358	
	Inclination of upper lip	120	7.94	2.00	238.00	<0.001	***	0.04	-2.0	0.7	0.009	**	-0.3	0.6	1.000		-2.3	0.7	0.002	**
	Inclination of lower lip	120	3.36	1.77	210.32	0.042	*	0.01	2.1	0.9	0.059		-0.7	0.7	0.860		1.4	0.9	0.375	
	Upper lip height-mouth width index	116	15.99	1.84	211.59	<0.001	***	0.08	1.0	0.6	0.221		1.9	0.4	<0.001	***	2.9	0.5	<0.001	***
	Mouth width contour index	117	3.17	1.85	214.40	0.048	*	0.01	0.1	0.1	0.191		0.0	0.0	1.000		0.1	0.1	0.096	
	Philtrum-mouth width index	117	3.32	2.00	232.00	0.038	*	0.01	0.7	0.3	0.045	*	-0.1	0.3	1.000		0.6	0.3	0.150	
	Medial-lateral cutaneous upper lip height index	116	2.65	1.77	203.99	0.080		0.01	-1.4	1.1	0.576		-0.9	0.8	0.814		-2.3	1.1	0.124	
	Vermilion-total upper lip height index	120	7.85^†^	..	..	0.020	*	..	1.5	..	0.032	*	0.2	..	0.980		1.7	..	0.053	
	Vermilion height index	120	20.62^†^	..	..	<0.001	***	..	3.0	..	0.147		2.4	..	0.022	*	5.4	..	<0.001	***
	Upper vermilion contour index	117	56.63^†^	..	..	<0.001	***	..	2.2	..	<0.001	***	1.4	..	0.002	**	3.7	..	<0.001	***
	Lower vermilion contour index	117	22.25	1.70	196.70	<0.001	***	0.11	0.7	0.3	0.082		1.2	0.2	<0.001	***	1.9	0.3	<0.001	***
	Lower-upper lip height index	120	21.72^†^	..	..	<0.001	***	..	-1.4	..	0.679		-2.7	..	<0.001	***	-4.2	..	0.001	**
	Cutaneous lower-upper lip height index	120	2.07^†^	..	..	0.356		..	3.2	..	0.638		-3.5	..	0.330		-0.2	..	0.863	
	Vermilion-total lower lip height index	120	1.22^†^	..	..	0.544		..	-1.6	..	0.795		0.1	..	0.894		-1.5	..	0.516	
	Vermilion arch index	117	7.50^†^	..	..	0.023	*	..	1.6	..	0.672		1.1	..	0.160		2.7	..	0.020	*
Cross-regional																				
	Upper face height-biocular width index	116	16.05^†^	..	..	<0.001	***	..	1.0	..	0.890		0.9	..	0.004	**	1.9	..	<0.001	***
	Biocular-face width index	117	59.09	1.57	182.35	<0.001	***	0.25	0.3	0.1	0.190		1.2	0.1	<0.001	***	1.4	0.2	<0.001	***
	Intercanthal width-upper face height index	116	11.18	1.52	174.67	<0.001	***	0.06	0.0	0.3	1.000		-1.0	0.2	<0.001	***	-1.0	0.3	<0.001	***
	Intercanthal-nasal width index	117	10.19	1.66	192.55	<0.001	***	0.05	1.0	0.4	0.043	*	0.6	0.3	0.074		1.5	0.4	<0.001	***
	Intercanthal-mouth width index	117	2.62	1.85	214.96	0.079		0.01	0.5	0.5	0.903		0.6	0.4	0.464		1.2	0.6	0.126	
	Nose-face width index	117	2.96	1.58	182.86	0.067		0.01	0.0	0.1	1.000		0.2	0.1	0.024	*	0.2	0.1	0.162	
	Nose-mouth width index	117	0.22	1.87	216.44	0.785		0.00	-0.3	0.5	1.000		0.2	0.4	1.000		-0.2	0.6	1.000	
	Nose height-face width index	116	17.23	1.40	161.10	<0.001	***	0.09	0.1	0.2	1.000		0.9	0.1	<0.001	***	1.1	0.2	<0.001	***
	Nose-face height index	120	18.29	1.72	205.07	<0.001	***	0.09	-0.6	0.1	<0.001	***	0.0	0.1	1.000		-0.7	0.1	<0.001	***
	Nose-upper face height index	120	21.39	1.68	200.07	<0.001	***	0.10	-0.5	0.2	0.004	**	-0.4	0.1	<0.001	***	-0.9	0.2	<0.001	***
	Nose-lower face height index	120	18.01	1.70	202.66	<0.001	***	0.09	-2.0	0.4	<0.001	***	-0.1	0.3	1.000		-2.0	0.4	<0.001	***
	Nasal tip protrusion-upper lip height index	120	32.00	1.67	198.28	<0.001	***	0.15	-2.0	0.7	0.018	*	-3.0	0.5	<0.001	***	-5.0	0.7	<0.001	***
	Mouth-face width index	117	1.04	1.88	217.59	0.351		0.00	0.2	0.2	1.000		0.1	0.2	1.000		0.3	0.2	0.591	
	Upper lip-upper face height index	120	21.39	1.68	200.07	<0.001	***	0.10	0.5	0.2	0.004	**	0.4	0.1	<0.001	***	0.9	0.2	<0.001	***
	Upper lip-mandible height index	120	6.44	1.73	205.78	0.003	**	0.03	-0.3	0.4	1.000		1.4	0.3	<0.001	***	1.1	0.5	0.044	*
	Upper lip-nose height index	120	20.83	1.70	202.28	<0.001	***	0.10	1.1	0.3	0.003	**	0.8	0.2	0.001	**	1.9	0.3	<0.001	***
	Lower lip-face height index	120	5.86	1.57	187.04	0.007	**	0.03	-0.2	0.2	1.000		-0.5	0.1	0.002	**	-0.7	0.2	0.009	**
	Lower lip-mandible height index	120	2.23	1.64	195.48	0.120		0.01	-0.4	0.4	1.000		-0.4	0.3	0.384		-0.8	0.4	0.178	
	Lower lip-chin height index	120	9.32^†^	..	..	0.009	**	..	-2.5	..	0.437		-0.4	..	0.167		-2.9	..	0.007	**

***p < 0.001.

**p < 0.01.

*p < 0.05.

*n* = sample size; *F* = *F*-value; *df*_*M*_ = model degree of freedom; *df*_*E*_ = error degree of freedom; *ES* = effect size; $\Delta \bar{x}$ = mean difference (measurement at the younger age was subtracted from the same measurement at the older age); *SE* = standard error.

†: due to non-normality of the data, Friedman test statistic was used to derive the *F*-value. Pairwise median differences were reported and statistically tested using Nemenyi post-hoc test.

### Growth changes in parameters for the overall face

Linear measurements: width of the face showed a significant increase of 2.0 mm in females (p < 0.001). Overall increase in width of the mandible was observed in both genders (females: 2.5 mm, p < 0.001; males: 1.5 mm, p = 0.041). All vertical measurements increased in both genders (p < 0.05) except for female height of the lower profile.

Angular measurements: in females, angle of total facial convexity was the only angular measurement that did not change with age (p = 0.359). In males, the only angular measurement that remained stable was the angle of facial convexity (p = 0.541). Mentocervical angle had an overall increase of 1.8° in females (p < 0.001) in contrast to an overall decrease of 1.7° driven by the significant decrease from 12 to 15 years in males (p < 0.001). Angle of the medium facial third decreased in both genders (females: -1.0°, p < 0.001; males: -1.0°, p < 0.001). Angle of the inferior facial third had an overall decrease of 1.6° in females (p < 0.001) and an increase of 0.6° in males (p = 0.021).

Profile inclinations: for females, all inclination measurements increased from 12 to 15 years of age (p < 0.001) but decreased subsequently from age 15 to 18 years (p < 0.001). In males, significant increases in inclination of the general profile line and inclination of the upper and lower face profile line were observed from 12 to 15 years (p < 0.05).

Proportion indices: for both genders, there was an overall increase in facial index (females: 1.6%, p < 0.001; males: 3.6%, p < 0.001). Decrease in mandible with-face height index was noted in both genders from 15 to 18 years (females: -1.7%, p = 0.014; males: -2.4%, p < 0.001). In the upper facial area, upper face index increased in both genders (females: 1.3%, p < 0.001; males: 2.2%, p < 0.001). In the lower facial area, mandibular index for males increased steadily over time by 1.2% (p = 0.004) while for females the index remained unchanged (p = 0.161). When upper and lower facial area was compared, mandible-face width index was found to increase only in males (1.2%, p = 0.002). In the chin area, chin-face height index and chin-mandible height index increased in both genders (p < 0.05). Comparing upper and lower chin area, chin index was found to decrease in males (6.9%, p = 0.014). In the mandibular area, mandibulo-face height index and mandibulo-upper face height index showed significant increase in males from 12 to 15 years (p < 0.05) and decrease in females from 15 to 18 years (p < 0.05). When mandible was investigated regionally in the lower facial area, both females and males were found to have decreased mandibulo-lower face height index (females: -0.9%, p < 0.001; males: -0.5%, p < 0.001). For indices of facial depth, upper-middle third face depth index became smaller in both genders (females: -0.7%, p = 0.010; males: -1.4%, p < 0.001). Middle-lower third face depth index had an overall increase of 0.4% (p = 0.001) in males while it remained unchanged in females (p = 0.327).

### Growth changes in parameters for the orbits

Linear measurements: all linear measurements investigated in the orbital region showed significant increase in both genders (p < 0.001). Notably, height of the eye fissure increased 11.1% in females and 9.8% in males.

Proportion indices: there was a general trend for increase in orbital width index in males (p < 0.001) but not in females (p = 0.491). In line with the significant increase in height of the eye fissure, eye fissure index increased steadily in both genders (females: 3.0%, p < 0.001; males: 1.6%, p < 0.001).

### Growth changes in parameters for the nose

Linear measurements: width of the nose increased 1.6% in females (0.6 mm, p < 0.001). A similar trend of increase was also observed in males (p = 0.020). Height of the nose increased 2.8% in females (1.3 mm, p < 0.001) and 3.0% in males (1.9 mm, p < 0.001). Length of the nasal bridge increased 1.1 mm in males (p = 0.002) and nasal protrusion increased 0.2 mm in females (p < 0.001).

Angular measurements: nasofrontal and nasomental angle decreased while nasofacial angle increased as a function of age in both genders (p < 0.001). Nasolabial angle revealed an overall increase in females (1.9°, p = 0.020) while no change was observed in males (p = 0.542).

Profile inclinations: Increased inclination of nasal bridge was observed in both females (1.5°, p < 0.001) and males (2.5°, p < 0.001).

Proportion indices: decrease of nasal index and nasal bridge index was observed in both genders (p < 0.05). A decrease in nasal tip protrusion-nose height index was observed in males (-0.8%, p < 0.001). Regarding the nostrils, nostril-nose width index and nostril width-nose height index revealed overall increase in both genders (p < 0.001).

### Growth changes in parameters for the lips and mouth

Linear measurements: philtrum widened 4.8% in females (0.5 mm, p < 0.001) and 3.4% in males (0.4 mm, p = 0.010). Changes in width of mouth was significant only in females (1.6 mm, p < 0.001). Height of the cutaneous lower lip did not change over time in either gender (p > 0.05). All other vertical measurements in the orolabial region increased in both genders (p < 0.05).

Angular measurements: no changes were observed for labiomental angle in either females (p = 0.556) or males (p = 0.094).

Profile inclinations: inclination of upper lip in males decreased progressively (p < 0.001). The lower lip in females became less oblique from age 12 to 15 years (3.7°, p < 0.001), but it grew more oblique from 15 to 18 years of age (-3.1°, p < 0.001).

Proportion indices: When upper lip was compared with lower lip, the lower-upper lip height index showed significant decrease in both genders (females: -2.0%, p < 0.001; males: -4.2%, p < 0.001). Vermilion height index was found to increase in males (5.4%, p < 0.001) while cutaneous lower-upper lip height index showed no age-related changes in either gender (p > 0.05). Within the upper lip area, vermilion-total upper lip height index increased in males from 12 to 15 years (1.5%, p = 0.032) but revealed stability in females (p = 0.770). Within the lower lip area, vermilion-total lower lip height index remained constant in both genders (p > 0.05).

### Growth changes in parameters for the cross-regional facial proportions

Cross-regional facial proportions demonstrated complex temporal changes. Of the 19 measurements investigated, 17 and 14 changed as a function of age in females and males, respectively. There were 10 measurements in females in which the direction of change from age 12 to 15 years was opposite to the direction of change from 15 to 18 years of age. In general, nasal height became proportionally smaller compared to upper, lower, and total facial height in both genders (p < 0.05). Upper lip became proportionally longer compared to mandible, nasal, and facial height in both genders (p < 0.05). Lower lip in males became proportionally shorter compared to facial and chin height (p < 0.05).

### Comparisons of growth changes of facial parameters between females and males

The amount of growth changes differ by gender (Tables 3, 4 and 5). The most remarkable gender difference was noted in nasofrontal angle (effect size: 0.55) and lower vermilion contour index (effect size: 0.59) from age 12 to 18 years. Further investigation revealed medium magnitude [45] of gender difference for nasofrontal angle (effect size: 0.43) from 12 to 15 years and for lower vermilion contour index from 12 to 15 years (effect size: 0.35) and from 15 to 18 years (effect size: 0.40).

**Table 3:** Comparison of growth changes of females and males from age 12 to 15 years.

Measurement		$\Delta \bar{x}$	*SE*	*LCI*	*UCI*	*p*-value		*ES*
Face								
	Width of the face	-1.4^†^	..	..	..	<0.001	***	0.23
	Width of the mandible	0.1^†^	..	..	..	0.944		0.00
	Height of the face	1.5^†^	..	..	..	0.328		0.06
	Height of the upper face	0.3^†^	..	..	..	0.697		0.02
	Height of the lower face	1.2^†^	..	..	..	0.180		0.08
	Height of the mandible	0.9^†^	..	..	..	0.224		0.07
	Height of the chin	0.4^†^	..	..	..	0.723		0.02
	Height of the lower profile	0.2^†^	..	..	..	0.826		0.01
	Height of the midface	-0.8^†^	..	..	..	0.124		0.09
	Lower half of the craniofacial height (left)	1.6	0.4	0.9	2.4	<0.001	***	0.27
	Lower half of the craniofacial height (right)	1.7	0.3	1.1	2.4	<0.001	***	0.30
	Mentocervical angle	-2.6	0.8	-4.1	-1.0	0.001	**	0.21
	Angle of facial convexity	-0.6^†^	..	..	..	0.128		0.09
	Angle of total facial convexity	-1.0	0.3	-1.6	-0.3	0.003	**	0.20
	Angle of the medium facial third	0.1^†^	..	..	..	0.738		0.02
	Angle of the inferior facial third	0.4^†^	..	..	..	0.034	*	0.13
	Inclination of general profile line	-1.5	0.5	-2.5	-0.5	0.003	**	0.18
	Inclination of upper face profile line	-1.2	0.5	-2.3	-0.2	0.019	*	0.15
	Inclination of lower face profile line	-1.8	0.6	-2.9	-0.7	0.001	**	0.20
	Inclination of lower third face line	-2.2^†^	..	..	..	0.001	**	0.20
	Inclination of the chin	-2.6^†^	..	..	..	<0.001	***	0.21
	Facial index	1.6^†^	..	..	..	0.021	*	0.14
	Mandible-face width index	0.7	0.4	-0.2	1.5	0.136		0.09
	Upper face index	0.8^†^	..	..	..	0.115		0.10
	Mandible width-face height index	-2.6^†^	..	..	..	0.297		0.07
	Mandibular index	1.7^†^	..	..	..	0.110		0.10
	Upper face-face height index	-0.4^†^	..	..	..	0.127		0.09
	Lower face-face height index	0.3^†^	..	..	..	0.029	*	0.13
	Chin-face height index	0.0	0.3	-0.5	0.4	0.853		0.01
	Chin-mandible height index	-0.7^†^	..	..	..	0.241		0.07
	Chin index	-2.5^†^	..	..	..	0.044	*	0.12
	Mandibulo-face height index	0.4^†^	..	..	..	0.127		0.09
	Mandibulo-upper face height index	1.1^†^	..	..	..	0.134		0.09
	Mandibulo-lower face height index	0.1	0.3	-0.5	0.6	0.759		0.02
	Mandible width-lower third face depth index	-1.9^†^	..	..	..	0.086		0.11
	Upper face height-upper third face depth index	-0.3	0.7	-1.7	1.0	0.612		0.03
	Mandible height-lower third face depth index	-0.3^†^	..	..	..	0.910		0.01
	Upper-middle third face depth index	-0.5^†^	..	..	..	0.015	*	0.15
	Middle-lower third face depth index	0.1	0.5	-0.8	1.0	0.760		0.02
	Upper cheek-upper third face depth index	-0.6	0.4	-1.3	0.1	0.101		0.10
Orbits								
	Intercanthal width	0.1^†^	..	..	..	0.291		0.06
	Biocular width	0.0^†^	..	..	..	0.653		0.03
	Length of the eye fissure (left)	-0.1^†^	..	..	..	0.914		0.01
	Length of the eye fissure (right)	0.1	0.1	-0.2	0.3	0.561		0.04
	Height of the eye fissure (left)	-0.2	0.1	-0.4	0.1	0.223		0.08
	Height of the eye fissure (right)	-0.3	0.1	-0.5	0.0	0.027	*	0.14
	Intercanthal index	0.0^†^	..	..	..	0.806		0.02
	Orbital width index	-0.1^†^	..	..	..	0.764		0.02
	Eye fissure index	-0.6	0.5	-1.6	0.3	0.203		0.08
Nose								
	Width of the nose	0.1	0.2	-0.3	0.4	0.773		0.02
	Height of the nose	0.3^†^	..	..	..	0.889		0.01
	Length of the nasal bridge	-0.1^†^	..	..	..	0.391		0.05
	Nasal protrusion	0.1^†^	..	..	..	0.925		0.01
	Nasofrontal angle	-3.1^†^	..	..	..	<0.001	***	0.43
	Nasal tip angle	-3.0^†^	..	..	..	<0.001	***	0.33
	Nasolabial angle	-1.8^†^	..	..	..	0.330		0.06
	Nasofacial angle	1.0^†^	..	..	..	<0.001	***	0.36
	Nasomental angle	-1.6^†^	..	..	..	<0.001	***	0.34
	Inclination of nasal bridge	-0.3	0.5	-1.2	0.6	0.579		0.03
	Nasal index	0.7^†^	..	..	..	0.978		0.00
	Nostril-nose width index	-1.4	0.5	-2.3	-0.4	0.005	**	0.17
	Nostril width-nose height index	-0.3^†^	..	..	..	0.107		0.10
	Nasal tip protrusion-width index	0.7^†^	..	..	..	0.034	*	0.13
	Nasal tip protrusion-nostril floor width index	2.5	0.9	0.7	4.2	0.007	**	0.17
	Nasal tip protrusion-nose height index	0.1	0.2	-0.4	0.5	0.748		0.02
	Nasal bridge index	0.8^†^	..	..	..	0.003	**	0.18
Lips and mouth								
	Width of the philtrum	0.4	0.2	0.0	0.7	0.039	*	0.13
	Width of the mouth	-1.1	0.4	-1.9	-0.2	0.013	*	0.15
	Height of the upper lip	0.5^†^	..	..	..	0.334		0.06
	Height of the cutaneous upper lip	-0.1	0.2	-0.6	0.4	0.729		0.02
	Vermilion height of the upper lip	0.6^†^	..	..	..	0.068		0.11
	Vermilion height of the lower lip	0.3^†^	..	..	..	0.221		0.08
	Height of the cutaneous lower lip	0.2	0.2	-0.3	0.7	0.420		0.05
	Height of the lower lip	0.4^†^	..	..	..	0.214		0.08
	Labiomental angle	1.1	1.3	-1.5	3.8	0.411		0.05
	Inclination of upper lip	-3.0^†^	..	..	..	0.006	**	0.17
	Inclination of lower lip	-1.6	1.2	-3.9	0.7	0.176		0.08
	Upper lip height-mouth width index	1.7	0.8	0.1	3.3	0.037	*	0.13
	Mouth width contour index	-0.1	0.1	-0.2	0.1	0.912		0.01
	Philtrum-mouth width index	1.5^†^	..	..	..	<0.001	***	0.22
	Medial-lateral cutaneous upper lip height index	0.2	1.6	-2.9	3.3	0.879		0.01
	Vermilion-total upper lip height index	1.9^†^	..	..	..	0.454		0.05
	Vermilion height index	1.0^†^	..	..	..	0.234		0.07
	Upper vermilion contour index	-1.9	0.6	-3.0	-0.8	<0.001	***	0.20
	Lower vermilion contour index	-2.1^†^	..	..	..	<0.001	***	0.35
	Lower-upper lip height index	0.8^†^	..	..	..	0.552		0.04
	Cutaneous lower-upper lip height index	1.7^†^	..	..	..	0.341		0.06
	Vermilion-total lower lip height index	-0.6	1.0	-2.6	1.4	0.570		0.04
	Vermilion arch index	0.6	0.8	-0.9	2.2	0.416		0.05
Cross-regional								
	Upper face height-biocular width index	0.2	0.7	-1.2	1.5	0.802		0.02
	Biocular-face width index	0.9^†^	..	..	..	<0.001	***	0.26
	Intercanthal width-upper face height index	-0.1^†^	..	..	..	0.830		0.01
	Intercanthal-nasal width index	-0.1	0.5	-1.0	0.8	0.789		0.02
	Intercanthal-mouth width index	2.2	0.8	0.7	3.8	0.005	**	0.17
	Nose-face width index	0.2^†^	..	..	..	0.051		0.12
	Nose-mouth width index	2.4	0.7	0.9	3.8	0.002	**	0.19
	Nose height-face width index	0.5^†^	..	..	..	0.215		0.08
	Nose-face height index	-0.3^†^	..	..	..	0.029	*	0.13
	Nose-upper face height index	-0.3	0.2	-0.7	0.1	0.178		0.08
	Nose-lower face height index	-0.9^†^	..	..	..	0.032	*	0.13
	Nasal tip protrusion-upper lip height index	-0.8	0.9	-2.6	1.0	0.395		0.05
	Mouth-face width index	-0.4	0.3	-1.0	0.1	0.131		0.09
	Upper lip-upper face height index	0.3	0.2	-0.1	0.7	0.178		0.08
	Upper lip-mandible height index	-0.2	0.6	-1.5	1.0	0.712		0.02
	Upper lip-nose height index	0.6	0.4	-0.2	1.5	0.146		0.09
	Lower lip-face height index	0.3^†^	..	..	..	0.712		0.02
	Lower lip-mandible height index	0.3^†^	..	..	..	0.951		0.00
	Lower lip-chin height index	1.2^†^	..	..	..	0.595		0.03

***p < 0.001.

**p < 0.01.

*p < 0.05.

$\Delta \bar{x}$ = mean difference (the magnitude of measurement change for females was subtracted from the magnitude of change for males. The magnitude of measurement change was estimated by subtracting measurement at the younger age from the same measurement at the older age); *SE* = standard error; *LCI* = lower limit of the 95% confidence interval; *UCI* = upper limit of the 95% confidence interval; *ES* = effect size.

†: due to non-normality of the data, median difference in the magnitude of measurement change from age 12 to 15 years between females and males was calculated and Mann-Whitney U test was used to derive the corresponding *p*-value.

**Table 4:** Comparison of growth changes of females and males from age 15 to 18 years.

Measurement		$\Delta \bar{x}$	*SE*	*LCI*	*UCI*	*p*-value		ES
Face								
	Width of the face	0.0	0.6	-1.2	1.1	0.933		0.01
	Width of the mandible	-0.2	0.8	-1.8	1.3	0.755		0.02
	Height of the face	0.8^†^	..	..	..	0.029	*	0.13
	Height of the upper face	0.6^†^	..	..	..	0.144		0.09
	Height of the lower face	0.1^†^	..	..	..	0.386		0.05
	Height of the mandible	0.6^†^	..	..	..	0.171		0.08
	Height of the chin	0.4^†^	..	..	..	0.426		0.05
	Height of the lower profile	1.1^†^	..	..	..	0.010	**	0.16
	Height of the midface	0.6^†^	..	..	..	0.453		0.05
	Lower half of the craniofacial height (left)	0.4	0.5	-0.5	1.3	0.436		0.05
	Lower half of the craniofacial height (right)	-0.1	0.5	-1.0	0.8	0.898		0.01
	Mentocervical angle	-0.7	0.6	-1.9	0.5	0.263		0.07
	Angle of facial convexity	1.0^†^	..	..	..	<0.001	***	0.24
	Angle of total facial convexity	0.4	0.2	0.0	0.7	0.039	*	0.13
	Angle of the medium facial third	0.3^†^	..	..	..	0.172		0.08
	Angle of the inferior facial third	1.5^†^	..	..	..	<0.001	***	0.45
	Inclination of general profile line	1.9	0.5	0.9	2.8	<0.001	***	0.24
	Inclination of upper face profile line	1.4	0.5	0.5	2.4	0.004	**	0.17
	Inclination of lower face profile line	2.4	0.5	1.5	3.4	<0.001	***	0.30
	Inclination of lower third face line	2.8^†^	..	..	..	<0.001	***	0.24
	Inclination of the chin	1.7^†^	..	..	..	0.003	**	0.18
	Facial index	0.3^†^	..	..	..	0.309		0.06
	Mandible-face width index	-0.2	0.4	-1.0	0.7	0.681		0.03
	Upper face index	0.3^†^	..	..	..	0.527		0.04
	Mandible width-face height index	-0.5	0.6	-1.8	0.8	0.429		0.05
	Mandibular index	0.3	0.3	-0.3	0.9	0.359		0.06
	Upper face-face height index	-0.1	0.2	-0.4	0.2	0.541		0.04
	Lower face-face height index	-0.1	0.1	-0.3	0.2	0.482		0.04
	Chin-face height index	0.2^†^	..	..	..	0.667		0.03
	Chin-mandible height index	0.0	0.4	-0.7	0.8	0.957		0.00
	Chin index	-4.7^†^	..	..	..	0.004	**	0.18
	Mandibulo-face height index	0.1	0.2	-0.2	0.4	0.541		0.04
	Mandibulo-upper face height index	0.3	0.4	-0.5	1.1	0.504		0.04
	Mandibulo-lower face height index	0.3	0.2	-0.1	0.7	0.191		0.08
	Mandible width-lower third face depth index	0.4	0.7	-1.0	1.8	0.596		0.03
	Upper face height-upper third face depth index	0.0^†^	..	..	..	0.723		0.02
	Mandible height-lower third face depth index	0.5^†^	..	..	..	0.043	*	0.12
	Upper-middle third face depth index	-0.1^†^	..	..	..	0.888		0.01
	Middle-lower third face depth index	1.0	0.3	0.5	1.6	<0.001	***	0.21
	Upper cheek-upper third face depth index	-0.3	0.3	-0.8	0.2	0.236		0.07
Orbits								
	Intercanthal width	0.4	0.1	0.1	0.6	0.006	**	0.17
	Biocular width	1.2	0.3	0.6	1.8	<0.001	***	0.22
	Length of the eye fissure (left)	0.7	0.1	0.4	0.9	<0.001	***	0.33
	Length of the eye fissure (right)	0.2	0.1	-0.1	0.5	0.139		0.09
	Height of the eye fissure (left)	0.0	0.1	-0.2	0.3	0.840		0.01
	Height of the eye fissure (right)	0.1	0.1	-0.1	0.4	0.249		0.07
	Intercanthal index	-0.2^†^	..	..	..	0.045	*	0.12
	Orbital width index	1.1^†^	..	..	..	<0.001	***	0.25
	Eye fissure index	-0.8	0.4	-1.7	0.1	0.067		0.11
Nose								
	Width of the nose	-0.2	0.2	-0.5	0.2	0.305		0.06
	Height of the nose	0.5^†^	..	..	..	0.032	*	0.13
	Length of the nasal bridge	0.2^†^	..	..	..	0.143		0.09
	Nasal protrusion	-0.3^†^	..	..	..	0.006	**	0.17
	Nasofrontal angle	-1.3^†^	..	..	..	<0.001	***	0.25
	Nasal tip angle	-0.7	0.4	-1.5	0.1	0.103		0.10
	Nasolabial angle	0.4^†^	..	..	..	0.665		0.03
	Nasofacial angle	-0.6	0.1	-0.9	-0.3	<0.001	***	0.26
	Nasomental angle	0.8	0.2	0.5	1.2	<0.001	***	0.28
	Inclination of nasal bridge	1.2	0.4	0.4	2.1	0.004	**	0.18
	Nasal index	-0.9	0.4	-1.7	0.0	0.040	*	0.13
	Nostril-nose width index	1.0	0.5	0.0	2.1	0.044	*	0.12
	Nostril width-nose height index	0.5	0.3	-0.1	1.1	0.091		0.11
	Nasal tip protrusion-width index	0.2	0.3	-0.3	0.7	0.423		0.05
	Nasal tip protrusion-nostril floor width index	-0.3	0.7	-1.7	1.0	0.621		0.03
	Nasal tip protrusion-nose height index	-0.7	0.2	-1.0	-0.4	<0.001	***	0.26
	Nasal bridge index	-0.2	0.2	-0.7	0.3	0.458		0.05
Lips and mouth								
	Width of the philtrum	-0.5	0.2	-0.8	-0.1	0.006	**	0.17
	Width of the mouth	0.0	0.4	-0.8	0.8	0.988		0.00
	Height of the upper lip	0.0^†^	..	..	..	0.915		0.01
	Height of the cutaneous upper lip	-0.2^†^	..	..	..	0.789		0.02
	Vermilion height of the upper lip	0.0^†^	..	..	..	0.877		0.01
	Vermilion height of the lower lip	-0.2^†^	..	..	..	0.139		0.09
	Height of the cutaneous lower lip	0.0^†^	..	..	..	0.954		0.00
	Height of the lower lip	-0.3	0.2	-0.6	0.0	0.069		0.11
	Labiomental angle	0.2	1.1	-1.9	2.4	0.841		0.01
	Inclination of upper lip	1.8^†^	..	..	..	0.082		0.11
	Inclination of lower lip	2.3^†^	..	..	..	0.004	**	0.18
	Upper lip height-mouth width index	-0.1^†^	..	..	..	0.726		0.02
	Mouth width contour index	0.0	0.1	-0.1	0.1	0.77		0.04
	Philtrum-mouth width index	-1.2	0.4	-1.9	-0.4	0.002	**	0.19
	Medial-lateral cutaneous upper lip height index	-5.2	1.2	-7.5	-2.8	<0.001	***	0.26
	Vermilion-total upper lip height index	0.6^†^	..	..	..	0.870		0.01
	Vermilion height index	2.2^†^	..	..	..	0.204		0.08
	Upper vermilion contour index	-0.4	0.4	-1.3	0.5	0.393		0.05
	Lower vermilion contour index	-2.1	0.3	-2.8	-1.5	<0.001	***	0.40
	Lower-upper lip height index	-0.3^†^	..	..	..	0.377		0.05
	Cutaneous lower-upper lip height index	-0.2^†^	..	..	..	0.882		0.01
	Vermilion-total lower lip height index	-0.6^†^	..	..	..	0.573		0.03
	Vermilion arch index	2.0	0.6	0.7	3.2	0.003	**	0.18
Cross-regional								
	Upper face height-biocular width index	-0.9	0.4	-1.6	-0.2	0.017	*	0.15
	Biocular-face width index	0.8	0.1	0.6	1.1	<0.001	***	0.39
	Intercanthal width-upper face height index	0.4	0.2	-0.1	0.9	0.084		0.11
	Intercanthal-nasal width index	1.5^†^	..	..	..	<0.001	***	0.25
	Intercanthal-mouth width index	0.7	0.6	-0.6	2.0	0.286		0.07
	Nose-face width index	-0.1^†^	..	..	..	0.198		0.08
	Nose-mouth width index	-0.5	0.6	-1.7	0.7	0.398		0.05
	Nose height-face width index	0.2^†^	..	..	..	0.256		0.07
	Nose-face height index	0.1	0.1	-0.2	0.3	0.482		0.04
	Nose-upper face height index	0.3	0.1	0.0	0.5	0.082		0.11
	Nose-lower face height index	0.3	0.4	-0.5	1.1	0.489		0.04
	Nasal tip protrusion-upper lip height index	-0.7	0.7	-2.0	0.6	0.285		0.07
	Mouth-face width index	0.0	0.3	-0.5	0.5	0.896		0.01
	Upper lip-upper face height index	-0.3	0.1	-0.5	0.0	0.082		0.11
	Upper lip-mandible height index	-0.5^†^	..	..	..	0.150		0.09
	Upper lip-nose height index	-0.7^†^	..	..	..	0.104		0.10
	Lower lip-face height index	-0.5	0.2	-1.0	-0.1	0.018	*	0.15
	Lower lip-mandible height index	-1.0	0.4	-1.7	-0.3	0.009	**	0.16
	Lower lip-chin height index	-1.6	1.1	-3.8	0.6	0.155		0.09

***p < 0.001.

**p < 0.01.

*p < 0.05.

$\Delta \bar{x}$ = mean difference (the magnitude of measurement change for females was subtracted from the magnitude of change for males. The magnitude of measurement change was estimated by subtracting measurement at the younger age from the same measurement at the older age); *SE* = standard error; *LCI* = lower limit of the 95% confidence interval; *UCI* = upper limit of the 95% confidence interval; *ES* = effect size.

†: due to non-normality of the data, median difference in the magnitude of measurement change from age 15 to 18 years between females and males was calculated and Mann-Whitney U test was used to derive the corresponding *p*-value.

**Table 5:** Comparison of growth changes of females and males from age 12 to 18 years.

Measurement		$\Delta \bar{x}$	*SE*	*LCI*	*UCI*	*p*-value		*ES*
Face								
	Width of the face	-1.1^†^	..	..	..	0.025	*	0.14
	Width of the mandible	-0.3	0.9	-2.1	1.4	0.710		0.02
	Height of the face	1.9^†^	..	..	..	0.113		0.10
	Height of the upper face	0.9^†^	..	..	..	0.295		0.06
	Height of the lower face	1.6^†^	..	..	..	0.103		0.10
	Height of the mandible	0.2^†^	..	..	..	0.145		0.09
	Height of the chin	-0.1^†^	..	..	..	0.703		0.02
	Height of the lower profile	1.0^†^	..	..	..	0.227		0.07
	Height of the midface	-0.6^†^	..	..	..	0.266		0.07
	Lower half of the craniofacial height (left)	2.0	0.5	0.9	3.1	<0.001	***	0.22
	Lower half of the craniofacial height (right)	1.7	0.5	0.6	2.7	0.002	**	0.19
	Mentocervical angle	-3.3	0.8	-4.9	-1.6	<0.001	***	0.26
	Angle of facial convexity	0.3^†^	..	..	..	0.326		0.06
	Angle of total facial convexity	-0.6	0.3	-1.3	0.1	0.085		0.12
	Angle of the medium facial third	0.1	0.2	-0.4	0.6	0.737		0.02
	Angle of the inferior facial third	1.9^†^	..	..	..	<0.001	***	0.44
	Inclination of general profile line	0.4	0.6	-0.8	1.5	0.518		0.04
	Inclination of upper face profile line	0.2	0.6	-1.0	1.4	0.742		0.02
	Inclination of lower face profile line	0.6	0.6	-0.6	1.9	0.322		0.06
	Inclination of lower third face line	-0.2	1.0	-2.1	1.7	0.835		0.01
	Inclination of the chin	0.3^†^	..	..	..	0.714		0.02
	Facial index	1.2^†^	..	..	..	0.015	*	0.15
	Mandible-face width index	0.5	0.5	-0.5	1.4	0.313		0.06
	Upper face index	0.9	0.4	0.0	1.7	0.049	*	0.12
	Mandible width-face height index	-1.3	1.1	-3.5	0.9	0.238		0.07
	Mandibular index	1.0	0.5	0.0	2.1	0.048	*	0.12
	Upper face-face height index	-0.1^†^	..	..	..	0.237		0.07
	Lower face-face height index	0.3	0.2	-0.1	0.6	0.135		0.09
	Chin-face height index	0.0	0.3	-0.5	0.5	0.929		0.01
	Chin-mandible height index	-0.4^†^	..	..	..	0.480		0.04
	Chin index	-8.6	1.9	-12.3	-4.8	<0.001	***	0.27
	Mandibulo-face height index	0.1^†^	..	..	..	0.237		0.07
	Mandibulo-upper face height index	0.2^†^	..	..	..	0.232		0.07
	Mandibulo-lower face height index	0.2^†^	..	..	..	0.302		0.06
	Mandible width-lower third face depth index	-1.5^†^	..	..	..	0.116		0.10
	Upper face height-upper third face depth index	-1.6^†^	..	..	..	0.092		0.10
	Mandible height-lower third face depth index	0.1^†^	..	..	..	0.363		0.06
	Upper-middle third face depth index	-0.4^†^	..	..	..	0.026	*	0.14
	Middle-lower third face depth index	1.2	0.4	0.3	2.0	0.008	**	0.16
	Upper cheek-upper third face depth index	-1.3^†^	..	..	..	0.001	**	0.20
Orbits								
	Intercanthal width	0.2^†^	..	..	..	0.066		0.11
	Biocular width	1.2	0.3	0.6	1.9	<0.001	***	0.22
	Length of the eye fissure (left)	0.4^†^	..	..	..	<0.001	***	0.27
	Length of the eye fissure (right)	0.3	0.1	0.0	0.6	0.059		0.12
	Height of the eye fissure (left)	-0.1	0.1	-0.4	0.1	0.336		0.06
	Height of the eye fissure (right)	-0.1	0.1	-0.4	0.1	0.315		0.07
	Intercanthal index	0.0^†^	..	..	..	0.349		0.06
	Orbital width index	0.7^†^	..	..	..	0.021	*	0.14
	Eye fissure index	-0.8^†^	..	..	..	0.018	*	0.14
Nose								
	Width of the nose	-0.1	0.2	-0.6	0.3	0.543		0.04
	Height of the nose	0.4^†^	..	..	..	0.389		0.05
	Length of the nasal bridge	1.0^†^	..	..	..	0.083		0.11
	Nasal protrusion	-0.2^†^	..	..	..	0.239		0.07
	Nasofrontal angle	-4.4^†^	..	..	..	<0.001	***	0.55
	Nasal tip angle	-3.4^†^	..	..	..	<0.001	***	0.37
	Nasolabial angle	-1.5^†^	..	..	..	0.092		0.10
	Nasofacial angle	1.0^†^	..	..	..	0.004	**	0.18
	Nasomental angle	-1.1^†^	..	..	..	0.006	**	0.17
	Inclination of nasal bridge	1.0	0.5	0.0	2.0	0.054		0.12
	Nasal index	-1.4^†^	..	..	..	0.123		0.10
	Nostril-nose width index	-0.3	0.6	-1.4	0.8	0.568		0.04
	Nostril width-nose height index	-0.4^†^	..	..	..	0.370		0.06
	Nasal tip protrusion-width index	1.3^†^	..	..	..	0.008	**	0.17
	Nasal tip protrusion-nostril floor width index	2.1	1.0	0.1	4.1	0.036	*	0.13
	Nasal tip protrusion-nose height index	-0.6	0.3	-1.1	-0.1	0.014	*	0.15
	Nasal bridge index	0.7	0.3	0.1	1.4	0.021	*	0.14
Lips and mouth								
	Width of the philtrum	-0.2^†^	..	..	..	0.477		0.04
	Width of the mouth	-0.4^†^	..	..	..	0.035	*	0.13
	Height of the upper lip	0.2	0.2	-0.3	0.7	0.438		0.05
	Height of the cutaneous upper lip	-0.2^†^	..	..	..	0.587		0.03
	Vermilion height of the upper lip	0.4^†^	..	..	..	0.100		0.10
	Vermilion height of the lower lip	-0.2^†^	..	..	..	0.537		0.04
	Height of the cutaneous lower lip	0.2	0.2	-0.3	0.6	0.512		0.04
	Height of the lower lip	-0.3^†^	..	..	..	0.683		0.03
	Labiomental angle	1.3	1.3	-1.3	3.9	0.316		0.06
	Inclination of upper lip	-1.3	0.9	-3.1	0.5	0.149		0.09
	Inclination of lower lip	0.8	1.2	-1.5	3.2	0.476		0.04
	Upper lip height-mouth width index	1.6	0.7	0.1	3.0	0.036	*	0.13
	Mouth width contour index	0.0	0.1	-0.1	0.2	0.909		0.01
	Philtrum-mouth width index	0.3^†^	..	..	..	0.437		0.05
	Medial-lateral cutaneous upper lip height index	-4.9	1.5	-7.8	-2.0	<0.001	***	0.21
	Vermilion-total upper lip height index	0.7	0.8	-0.8	2.2	0.354		0.06
	Vermilion height index	4.3^†^	..	..	..	0.046	*	0.12
	Upper vermilion contour index	-2.3	0.5	-3.3	-1.3	<0.001	***	0.27
	Lower vermilion contour index	-4.6	0.4	-5.3	-3.8	<0.001	***	0.59
	Lower-upper lip height index	0.0^†^	..	..	..	0.592		0.03
	Cutaneous lower-upper lip height index	2.0	2.1	-2.1	6.1	0.346		0.06
	Vermilion-total lower lip height index	-1.2^†^	..	..	..	0.461		0.05
	Vermilion arch index	2.6	0.7	1.2	4.1	<0.001	***	0.21
Cross-regional								
	Upper face height-biocular width index	-0.7^†^	..	..	..	0.529		0.04
	Biocular-face width index	1.5	0.2	1.1	1.9	<0.001	***	0.45
	Intercanthal width-upper face height index	0.3^†^	..	..	..	0.586		0.03
	Intercanthal-nasal width index	1.3	0.5	0.4	2.2	0.004	**	0.20
	Intercanthal-mouth width index	2.9	0.7	1.4	4.4	<0.001	***	0.23
	Nose-face width index	-0.1^†^	..	..	..	0.501		0.04
	Nose-mouth width index	1.8	0.7	0.4	3.3	0.010	*	0.16
	Nose height-face width index	0.8^†^	..	..	..	0.025	*	0.14
	Nose-face height index	-0.3	0.2	-0.6	0.1	0.135		0.09
	Nose-upper face height index	0.0	0.2	-0.4	0.4	0.934		0.01
	Nose-lower face height index	-0.8	0.6	-1.9	0.3	0.141		0.09
	Nasal tip protrusion-upper lip height index	-1.5	0.9	-3.3	0.3	0.108		0.10
	Mouth-face width index	-0.4	0.3	-1.0	0.1	0.148		0.09
	Upper lip-upper face height index	0.0	0.2	-0.4	0.4	0.934		0.01
	Upper lip-mandible height index	-0.4^†^	..	..	..	0.299		0.06
	Upper lip-nose height index	0.0	0.4	-0.8	0.9	0.912		0.01
	Lower lip-face height index	-0.8^†^	..	..	..	0.051		0.12
	Lower lip-mandible height index	-1.2^†^	..	..	..	0.022	*	0.14
	Lower lip-chin height index	-1.7^†^	..	..	..	0.251		0.07

***p < 0.001.

**p < 0.01.

*p < 0.05.

$\Delta \bar{x}$ = mean difference (the magnitude of measurement change for females was subtracted from the magnitude of change for males. The magnitude of measurement change was estimated by subtracting measurement at the younger age from the same measurement at the older age); *SE* = standard error; *LCI* = lower limit of the 95% confidence interval; *UCI* = upper limit of the 95% confidence interval; *ES* = effect size.

†: due to non-normality of the data, median difference in the magnitude of measurement change from age 12 to 18 years between females and males was calculated and Mann-Whitney U test was used to derive the corresponding *p*-value.

## Discussion

In this longitudinal population-representative photogrammetric study, growth changes in the face were noted in both genders. Nasofrontal angle and lower vermilion contour index demonstrated large magnitude of gender difference (effect size greater than 0.5, according to Cohen [45]’s criteria) in the amount of facial growth over the entire observation period. A discussion of our findings are provided in this section.

### Examiner reliability

The magnitude of ME is generally similar to the values reported in previous photogrammetric studies [48–50]. Since ME has been found to overestimate the true random error, we also reported MME, a distortion-free measure of random error [38]. For several measurements, the magnitude of ME and MME was larger than the amount of growth changes, a finding also noted in a longitudinal cephalometric study by Sarnäs and Solow [51]. While some caution needs to be exercised when interpreting these measurements, as Sarnäs and Solow [51] emphasized, this should not overshadow the importance of the present findings because they nevertheless provide indications of the magnitude and direction of the trend of growth.

### General patterns of facial growth

#### Growth in the overall face

Sforza et al. [52] investigated age-related changes in linear facial measurements of a Northern Sudanese population aged 4 to 30 years. All linear distances increased significantly with age in Sudanese. Consistent with Sforza et al.’s reports [52], our findings showed that all horizontal and vertical linear measurements increased steadily from 12 to 18 years of age except for height of the lower profile in females and width of the face in males.

Existing data on growth changes of angular and proportion measurements of the overall face are sparse except for angle of facial convexity and angle of total facial convexity. With longitudinal cephalometric radiographs of 15 girls and 14 boys, Vahdettin and Altuğ [53] reported that angle of facial convexity remained constant from age 10 to 16 years in both genders. The present study found that the angle was stable in males while it decreased in females from age 15 to 18 years. The different findings for females may result from continued growth changes after 16 years of age. Angle of total facial convexity in this study had no significant changes in females while it decreased in males. Confirming results from Vahdettin and Altuğ [53] and Ferrario et al. [54], our findings suggest that the nose moves forward to a larger degree in males than in females during growth.

Findings on proportion indices suggest that the upper face grow proportionally narrower and longer in both genders. Similar growth pattern was noted for the lower face in males. In females, while the lower face width (go-go) became proportionally narrower compared to the overall height of the face (n-me), no age-related change was observed when lower face width (go-go) was compared regionally to height of the mandible (sto-me). Lower face width (go-go) grew proportionally wider compared to the middle facial area (zy-zy) in males while in females the proportion remain unchanged. In the chin area, facial indices indicated a proportionally longer chin with growth in both genders. This observation is in line with findings of several studies analyzing growth changes of facial forms [55, 56]. In addition, it was found that the lower chin height (pg-me) grew proportionally larger compared to the upper chin height (sl-pg) in males. In the mandibular area, the mandible height (sto-me) grew proportionally longer in males but proportionally shorter in females relative to the overall (n-me) and upper facial height (n-sto). But regionally compared to the lower facial height (sn-me), the mandible height (sto-me) decreased proportionally in both genders. This reduction was attributable to the significant increase in upper lip height (sn-sto) in both genders. For facial depth indices, upper third facial depth (t-n) decreased proportionally relative to middle third facial depth (t-sn) in both genders, which may result from the forward movement of the upper lip area with growth [53]. Relative depth of the middle (t-sn) to lower (t-me) facial third increased in males during the overall observation period but was stable in females. Considering that the increase in males primarily takes place between age 15 to 18 years, a period when thickening of the upper lip is negligible in females but peaks in males [28, 57], it is reasonable to assume that the gender difference may stem from the rapid thickening of upper lip in males during the above period.

#### Growth in the orbital region

The most notable growth changes in the orbital region was the eye fissure height, as evidenced by the 10% increase in absolute magnitude from age 12 to 18 years and the steady increase in eye fissure index. These findings converge with the age-related changes reported by Farkas et al. [35] and Farkas [34]. In contrast with Sforza et al. [19]’s finding that no further lengthening of eye fissure was observed in Italian females after 12–13 years of age, the eye fissure length continued to increase from 12 to 18 years of age in our population.

#### Growth in the nasal region

Sforza et al. [17] reported that nasal height grew the fasted and nasal width grew the slowest in the nasal region. Their findings were confirmed by the larger percentage increase in nasal height (around 3%) than the increase in nasal width (around 1.6%) and the progressive decrease in nasal index in this study. In Western populations, females were reported to have concluded a large part of nasal growth by age 12 [58], while continued growth after age 12 was noted in Chinese population.

In both genders, nasofrontal angle decreased while nasal facial angle and inclination of nasal bridge increased as a function of age. These changes reflect that the nasal dorsum becomes more horizontally inclined during growth, rendering the nose a more forward position. These changes are in accord with findings from earlier cephalometric studies [10, 59, 60]. In addition, the larger age-related decrease in nasofrontal angle in males versus in females and the decrease in angle of total facial convexity in males but not in females indicate that the forward positioning of the nose is more significant in males than in females, in line with findings from Vahdettin and Altuğ [53].

Most studies on growth of nasolabial angle reported no significant age-related changes in males [53, 58, 61, 62]. Although a decreasing trend from 7 to 18 years was reported by Nanda et al. [28], no statistical analyses were performed in their study. Our finding adds evidence to the stability of nasolabial angle in males from 12 to 18 years. Growth changes of nasolabial angle in females have been studied both cross-sectionally [28, 30] and longitudinally [53, 58, 62] with different findings. But longitudinal studies [53, 58, 62] have demonstrated stability of nasolabial angle in females. Findings from the present investigation suggest an age-related increase in nasolabial angle in females. Possible explanations for the different finding in the present study may be the different ethnic population investigated and the small sample size in previous longitudinal cephalometric studies.

#### Growth in the orolabial region

In agreement with the polynomial regression curves from Sforza et al. [20]’s study, lip height and vermilion height increased during growth in both genders. Proportion indices in the present study suggest that the increase in upper lip height in males was primarily contributed by the increase in upper vermilion. No change was observed in width of the mouth in females and height of the cutaneous lower lip in either gender.

In a cross-sectional study on Northern Sudanese populations [30], labiomental angle decreased progressively from the youngest (4-year-old) to the oldest (30-year-old) age group in females, while the change was inconsistent in males from age 12 to 17 years. In the present study, the amount of age-related changes in the angle was not significant in view of the large variability. The high ME and MME for labiomental angle in this study support Nanda et al. [28]’s statement that the large observed variability was attributable to the random error associated with digitization and it would suffice to be aware that growth changes are small for labiomental angle.

### Clinical relevance

By excluding participants with facial disharmonies, we were able to establish norms [63] of facial growth for Chinese in Hong Kong. Such norms are of significance in forensic, clinical, and research settings. First, a thorough understanding of facial growth increases the probability of successful location of missing children. Prediction of facial growth could provide important clues in identification of missing children [9]. The established norms allowed us to predict facial growth with ethnicity-specific data so that we were able to achieve greater precision in growth prediction for Chinese in Hong Kong. Second, the established norms are of value in clinical diagnosis and treatment planning. Age 12 to 18 years is a period of active orthodontic treatment. The established norms will assist orthodontists in determining the optimal amount, duration, and timing of treatment. The norms will also be of value to maxillofacial and plastic surgeons in their treatment planning for young patients originating from south China. Third, the established norms serve as a reference dataset to be compared against growth data from other studies. Comparing our norms with growth data collected from other ethnicities provides insight into developmental origins of inter-ethnic facial variations. In addition, it would be of interest to compare patterns of facial growth between participants with and without malocclusion.

### Strengths and limitations

This study has a number of key strengths. First, as far as we are aware, this is the first large, population-representative longitudinal photogrammetric study of the face. Our findings therefore reflect the amount and direction of facial growth with high level of accuracy. Second, this is one of the most comprehensive anthropometric studies to date in terms of the type (linear and angular measurements, profile inclinations, and proportion indices) and number (108 in total) of facial parameters investigated. Third, effect sizes are reported whenever possible. This allows future studies to be compared with current results not just based on the p values, which are influenced by sample size, but also based on a more generally interpretable description of the size of an effect [44].

Several factors have to be acknowledged as potential limitations of the current report. First, this study is subject to limitations inherent in photogrammetry. Distance to the camera is different among various facial features, those closer to the camera, such as the nose, would have larger degree of magnification on the photographs than the more distant features, such as the eyes. However, the differences between facial measurements in this study and those from three-dimensional studies generally fall within a reasonable range [17, 19, 20]. In addition, except for the tragion point, all other landmarks on lateral photographs were digitized along the facial midline. Thus facial measurements from lateral photographs are mostly free from such distortions. Second, changes in head positioning would distort the facial measurements derived from the photographs [27]. However, participants in this study were instructed to assume natural head position during photographing, a position that has been reported to be reproducible over 5- [64] and 15-year [65] interval. Third, the study period is short. Since the onset of puberty takes place at around age 11 years in girls [66], this study may have failed to capture a certain amount of growth changes in girls during the first year of puberty. On the other hand, facial growth continues after age 18 years [51], thus the photogrammetric measurements at 18 years in this study should not be taken as the norms of adult facial features of Chinese in Hong Kong. Longitudinal studies with a longer period of follow-up on different populations are recommended in order to achieve a complete understanding of the timing, duration, amount, and direction of facial growth in various ethnicities/races.

## Conclusion

In conclusion, the most remarkable changes in the overall face are the significant increase in various horizontal and vertical linear measurements and the changes in their relative proportions. Changes in the orbital region are characterized by the dramatic increase in eye fissure height in both genders. With growth, nose assumed a more forward position due to the more horizontally inclined nasal dorsum, which is more evident in males. In the orolabial region, there was a significant increase in the proportion of upper vermilion height relative to the upper lip height in males. Differences in growth changes between genders were most evident for nasofrontal angle and lower vermilion contour index during the observation period.

The above findings are believed to be fairly accurate due to the large sample size, representative sample, and longitudinal design of this study. The growth data may benefit different clinical specialties and other nonclinical fields where facial growth are of interest.

## Supporting information

S1 Table Definitions of the anthropometric landmarks used. (DOCX)

S2 Table Definitions of standard anthropometric measurements used. (DOCX)

S3 Table Reliability of photogrammetric measurements. (DOCX)

S4 Table Original data for linear and angular facial measurements calculated from frontal images. (XLSX)

S5 Table Original data for linear and angular facial measurements calculated from lateral images. (XLSX)

S6 Table Original data for proportion indices calculated from frontal images. (XLSX)

S7 Table Original data for proportion indices calculated from lateral images. (XLSX)

S8 Table Original data for proportion indices calculated from both frontal and lateral images. (XLSX)

S9 Table Original data for inclination indices. (XLSX)
