# Supplementary material for: A longitudinal study of facial growth of Southern Chinese in Hong Kong: Comprehensive photogrammetric analyses
Source: PLoS One. 2017 Oct 20;12(10):e0186598. doi: 10.1371/journal.pone.0186598 (PMC5650157; doi:10.1371/journal.pone.0186598)
Supplement: S1 Table — (DOCX) [file pone.0186598.s001.docx]

**S1 Table. Definitions of the anthropometric landmarks used.**

| Landmark | | Definition |
| --- | --- | --- |
|  |  |  |
| Frontal images | |  |
|  | Endocanthion (en) | The point at the inner commissure of the eye fissure |
|  | Exocanthion (ex) | The point at the outer commissure of the eye fissure |
|  | Palpebrale superius (ps) | The highest point in the midportion of the free margin of each upper eyelid |
|  | Palpebrale inferius (pi) | The lowest point in the midportion of the free margin of each lower eyelid |
|  | Alare (al) | The most lateral point on each alar contour |
|  | Subalare (sbal) | The point at the lower limit of each alar base |
|  | Labrale superius (ls) | The midline point representing the mucocutaneous vermilion border of the upper lip |
|  | Crista philtri (cph) | The point on each elevated margin of the philtrum just above the vermilion line |
|  | Labrale superius' (ls') | The point on the upper vermilion line vertically beneath the subalare point |
|  | Cheilion (ch) | The point located at each lateral oral commissure |
|  | Stomion (sto) | The most anterior midline point of contact between the upper and lower lip |
|  | Labrale inferius (li) | The midline point representing the mucocutaneous vermilion border of the lower lip |
|  | Zygion (zy) | The most lateral soft tissue point overlying each zygomatic arch |
|  | Gonion (go) | The most lateral point on the mandibular angle |
|  | Menton (me) | The most inferior midline point of the soft tissue chin |
| Lateral images | |  |
|  | Glabella (g) | The most prominent midline point of the forehead between the brow ridges |
|  | Nasion (n) | The point in the midline of the nasal radix and nasofrontal region |
|  | Pronasale (prn) | The most prominent point on the nasal tip |
|  | Columella breakpoint (c') | The point at the mid-columella, where the columella takes a more horizontal course, extending posteriorly to subnasale |
|  | Subnasale (sn) | The deepest midline point where the base of the nasal columella meets the upper lip |
|  | Labrale superius (ls) | The midline point representing the mucocutaneous vermilion border of the upper lip |
| **S1 Table. Definitions of the anthropometric landmarks used (continued).** | | |
| Landmark | | Definition |
|  |  |  |
|  | Stomion (sto) | The most anterior midline point of contact between the upper and lower lip |
|  | Labrale inferius (li) | The midline point representing the mucocutaneous vermilion border of the lower lip |
|  | Sublabiale (sl) | The midline point of greatest concavity on the facial contour of the lower lip between labrale inferius and soft tissue menton |
|  | Pogonion (pg) | The most prominent midline point of the soft tissue chin pad |
|  | Menton (me) | The most inferior midline point of the soft tissue chin |
|  | Cervical point (c) | The innermost point between the submental region and the anterior surface of the neck, in the midsagittal plane |
|  | Tragion (t) | The notch in the superior margin of each tragus |
