# Supplementary material for: A longitudinal study of facial growth of Southern Chinese in Hong Kong: Comprehensive photogrammetric analyses
Source: PLoS One. 2017 Oct 20;12(10):e0186598. doi: 10.1371/journal.pone.0186598 (PMC5650157; doi:10.1371/journal.pone.0186598)
Supplement: S2 Table — (DOCX) [file pone.0186598.s002.docx]

**S2 Table. Definitions of standard anthropometric measurements used.**

| Measurement | |  | Definition | Image view |
| --- | --- | --- | --- | --- |
| Face | |  |  |  |
|  | Width of the face^a^ |  | $zy^{l}-zy^{r}$ | F |
|  | Width of the mandible^a^ |  | $go^{l}-go^{r}$ | F |
|  | Height of the face^b^ |  | $n-me$ | L |
|  | Height of the upper face^b^ |  | $n-sto$ | L |
|  | Height of the lower face^b^ |  | $sn-me$ | L |
|  | Height of the mandible^b^ |  | $sto-me$ | L |
|  | Height of the chin^b^ |  | $sl-me$ | L |
|  | Height of the lower profile^b^ |  | $prn-me$ | L |
|  | Height of the midface^b^ |  | $g-sn$ | L |
|  | Lower half of the craniofacial height (left)^b^ |  | $en^{l}-me$ | F |
|  | Lower half of the craniofacial height (right)^b^ |  | $en^{r}-me$ | F |
|  | Mentocervical angle |  | $\angle c-me/g-pg$ | L |
|  | Angle of facial convexity |  | $\angle g-sn-pg$ | L |
|  | Angle of total facial convexity |  | $\angle g-prn-pg$ | L |
|  | Angle of the medium facial third |  | $\angle n-t-sn$ | L |
|  | Angle of the inferior facial third |  | $\angle sn-t-me$ | L |
|  | Inclination of general profile line |  | $Inclination: g-pg$ | L |
|  | Inclination of upper face profile line |  | $Inclination: g-sn$ | L |
|  | Inclination of lower face profile line |  | $Inclination: sn-pg$ | L |
|  | Inclination of lower third face line |  | $Inclination: li-pg$ | L |
|  | Inclination of the chin |  | $Inclination: sl-pg$ | L |
|  | Facial index |  | $\frac{n-me}{zy^{l}-zy^{r}}\times100$ | F & L |
|  | Mandible-face width index |  | $\frac{go^{l}-go^{r}}{zy^{l}-zy^{r}}\times100$ | F |
|  | Upper face index |  | $\frac{n-sto}{zy^{l}-zy^{r}}\times100$ | F & L |
|  | Mandible width-face height index |  | $\frac{go^{l}-go^{r}}{n-me}\times100$ | F & L |
|  | Mandibular index |  | $\frac{sto-me}{go^{l}-go^{r}}\times100$ | F & L |
|  | Upper face-face height index |  | $\frac{n-sto}{n-me}\times100$ | L |
|  | Lower face-face height index |  | $\frac{sn-me}{n-me}\times100$ | L |
|  | Chin-face height index |  | $\frac{sl-me}{n-me}\times100$ | L |
|  | Chin-mandible height index |  | $\frac{sl-me}{sto-me}\times100$ | L |
|  | Chin index |  | $\frac{sl-pg}{pg-me}\times100$ | L |
|  | Mandibulo-face height index |  | $\frac{sto-me}{n-me}\times100$ | L |
|  | Mandibulo-upper face height index |  | $\frac{sto-me}{n-sto}\times100$ | L |
| **S2 Table. Definitions of standard anthropometric measurements used (continued).** | | | | |
| Measurement | |  | Definition | Image view |
|  | Mandibulo-lower face height index |  | $\frac{sto-me}{sn-me}\times100$ | L |
|  | Mandible width-lower third face depth index |  | $\frac{go^{l}-go^{r}}{me-t}\times100$ | F & L |
|  | Upper face height-upper third face depth index |  | $\frac{n-sto}{t-n}\times100$ | L |
|  | Mandible height-lower third face depth index |  | $\frac{sto-me}{t-me}\times100$ | L |
|  | Upper-middle third face depth index |  | $\frac{t-n}{t-sn}\times100$ | L |
|  | Middle-lower third face depth index |  | $\frac{t-sn}{t-me}\times100$ | L |
|  | Upper cheek-upper third face depth index |  | $\frac{t-ex^{r}}{t-n}\times100$ | L |
| Orbits | |  |  |  |
|  | Intercanthal width^a^ |  | $en^{l}-en^{r}$ | F |
|  | Biocular width^a^ |  | $ex^{l}-ex^{r}$ | F |
|  | Length of the eye fissure (left)^a^ |  | $ex^{l}-en^{l}$ | F |
|  | Length of the eye fissure (right)^a^ |  | $ex^{r}-en^{r}$ | F |
|  | Height of the eye fissure (left)^b^ |  | $ps^{l}-pi^{l}$ | F |
|  | Height of the eye fissure (right)^b^ |  | $ps^{r}-pi^{r}$ | F |
|  | Intercanthal index |  | $\frac{en^{l}-en^{r}}{ex^{l}-ex^{r}}\times100$ | F |
|  | Orbital width index |  | $\frac{ex^{l}-en^{l}}{en^{l}-en^{r}}\times100$ | F |
|  | Eye fissure index |  | $\frac{ps^{l}-pi^{l}}{ex^{l}-en^{l}}\times100$ | F |
| Nose | |  |  |  |
|  | Width of the nose^a^ |  | $al^{l}-al^{r}$ | F |
|  | Height of the nose^b^ |  | $n-sn$ | L |
|  | Length of the nasal bridge^a^ |  | $n-prn$ | L |
|  | Nasal protrusion^a^ |  | $sn-prn$ | L |
|  | Nasofrontal angle |  | $\angle g-n-prn$ | L |
|  | Nasal tip angle |  | $\angle n-prn/c'-sn$ | L |
|  | Nasolabial angle |  | $\angle c'-sn-ls$ | L |
|  | Nasofacial angle |  | $\angle g-pg/n-prn$ | L |
|  | Nasomental angle |  | $\angle n-prn-pg$ | L |
|  | Inclination of nasal bridge |  | $Inclination: n-prn$ | L |
|  | Nasal index |  | $\frac{al^{l}-al^{r}}{n-sn}\times100$ | F & L |
|  | Nostril-nose width index |  | $\frac{sbal^{l}-sn+sbal^{r}-sn}{al^{l}-al^{r}}\times100$ | F |
|  | Nostril width-nose height index |  | $\frac{sbal^{l}-sn}{n-sn}\times100$ | F & L |
|  | Nasal tip protrusion-width index |  | $\frac{sn-prn}{al^{l}-al^{r}}\times100$ | F & L |
| **S2 Table. Definitions of standard anthropometric measurements used (continued).** | | | | |
| Measurement | |  | Definition | Image view |
|  | Nasal tip protrusion-nostril floor width index |  | $\frac{sn-prn}{sbal^{l}-sn+sbal^{r}-sn}\times100$ | F & L |
|  | Nasal tip protrusion-nose height index |  | $\frac{sn-prn}{n-sn}\times100$ | L |
|  | Nasal bridge index |  | $\frac{n-prn}{n-sn}\times100$ | L |
| Lips and mouth | |  |  |  |
|  | Width of the philtrum^a^ |  | $cph^{l}-cph^{r}$ | F |
|  | Width of the mouth^a^ |  | $ch^{l}- ch^{r}$ | F |
|  | Height of the upper lip^b^ |  | $sn-sto$ | L |
|  | Height of the cutaneous upper lip^b^ |  | $sn-ls$ | L |
|  | Vermilion height of the upper lip^b^ |  | $ls-sto$ | L |
|  | Vermilion height of the lower lip^b^ |  | $sto-li$ | L |
|  | Height of the cutaneous lower lip^b^ |  | $li-sl$ | L |
|  | Height of the lower lip^b^ |  | $sto-sl$ | L |
|  | Labiomental angle |  | $\angle li-sl-pg$ | L |
|  | Inclination of upper lip |  | $Inclination: sn-ls$ | L |
|  | Inclination of lower lip |  | $Inclination: li-sl$ | L |
|  | Upper lip height-mouth width index |  | $\frac{sn-sto}{ch^{l}-ch^{r}}\times100$ | F & L |
|  | Mouth width contour index |  | $\frac{ch^{l}-ch^{r}}{ch^{l}-sto+ch^{r}-sto}\times100$ | F |
|  | Philtrum-mouth width index |  | $\frac{cph^{l}-cph^{r}}{ch^{l}-ch^{r}}\times100$ | F |
|  | Medial-lateral cutaneous upper lip height index |  | $\frac{sn-ls}{sbal^{l}-ls^{'l}}\times100$ | F & L |
|  | Vermilion-total upper lip height index |  | $\frac{ls-sto}{sn-sto}\times100$ | L |
|  | Vermilion height index |  | $\frac{ls-sto}{sto-li}\times100$ | L |
|  | Upper vermilion contour index |  | $\frac{ch^{l}-ch^{r}}{ch^{l}-ls-ch^{r}}\times100$ | F |
|  | Lower vermilion contour index |  | $\frac{ch^{l}-ch^{r}}{ch^{l}-li-ch^{r}}\times100$ | F |
|  | Lower-upper lip height index |  | $\frac{sto-sl}{sn-sto}\times100$ | L |
|  | Cutaneous lower-upper lip height index |  | $\frac{li-sl}{sn-ls}\times100$ | L |
|  | Vermilion-total lower lip height index |  | $\frac{sto-li}{sto-sl}\times100$ | L |
|  | Vermilion arch index |  | $\frac{ch^{l}-li-ch^{r}}{ch^{l}-ls-ch^{r}}\times100$ | F |
| Cross-regional | |  |  |  |
|  | Upper face height-biocular width index |  | $\frac{n-sto}{ex^{l}-ex^{r}}\times100$ | F & L |
|  | Biocular-face width index |  | $\frac{ex^{l}-ex^{r}}{zy^{l}-zy^{r}}\times100$ | F |
|  | Intercanthal width-upper face height index |  | $\frac{en^{l}-en^{r}}{n-sto} \times100$ | F & L |
| **S2 Table. Definitions of standard anthropometric measurements used (continued).** | | | | |
| Measurement | |  | Definition | Image view |
|  | Intercanthal-nasal width index |  | $\frac{en^{l}-en^{r}}{al^{l}-al^{r}}\times100$ | F |
|  | Intercanthal-mouth width index |  | $\frac{en^{l}-en^{r}}{ch^{l}-ch^{r}}\times100$ | F |
|  | Nose-face width index |  | $\frac{al^{l}-al^{r}}{zy^{l}-zy^{r}}\times100$ | F |
|  | Nose-mouth width index |  | $\frac{al^{l}-al^{r}}{ch^{l}-ch^{r}} \times100$ | F |
|  | Nose height-face width index |  | $\frac{n-sn}{zy^{l}-zy^{r}}\times100$ | F & L |
|  | Nose-face height index |  | $\frac{n-sn}{n-me}\times100$ | L |
|  | Nose-upper face height index |  | $\frac{n-sn}{n-sto}\times100$ | L |
|  | Nose-lower face height index |  | $\frac{n-sn}{sn-me}\times100$ | L |
|  | Nasal tip protrusion-upper lip height index |  | $\frac{sn-prn}{sn-sto}\times100$ | L |
|  | Mouth-face width index |  | $\frac{ch^{l}-ch^{r}}{zy^{l}-zy^{r}}\times100$ | F |
|  | Upper lip-upper face height index |  | $\frac{sn-sto}{n-sto}\times100$ | L |
|  | Upper lip-mandible height index |  | $\frac{sn-sto}{sto-me}\times100$ | L |
|  | Upper lip-nose height index |  | $\frac{sn-sto}{n-sn}\times100$ | L |
|  | Lower lip-face height index |  | $\frac{sto-sl}{sn-me}\times100$ | L |
|  | Lower lip-mandible height index |  | $\frac{sto-sl}{sto-me}\times100$ | L |
|  | Lower lip-chin height index |  | $\frac{sto-sl}{sl-me}\times100$ | L |

^l^The left of the bilaterally homologous landmarks; ^r^the right of the bilaterally homologous landmarks.

^a^The linear measurement represents point-to-point distance between landmarks; ^b^the linear measurement was calculated as the vertical distance between landmarks.

F: the measurement was taken on frontal photographs; L: the measurement was taken on lateral photographs; F & L: both frontal and lateral photographs were used to calculate the measurement.
