# Supplementary material for: A longitudinal study of facial growth of Southern Chinese in Hong Kong: Comprehensive photogrammetric analyses
Source: PLoS One. 2017 Oct 20;12(10):e0186598. doi: 10.1371/journal.pone.0186598 (PMC5650157; doi:10.1371/journal.pone.0186598)
Supplement: S3 Table — (DOCX) [file pone.0186598.s003.docx]

**S3 Table. Reliability of photogrammetric measurements.**

| Measurement | | ME | MME | ICC |
| --- | --- | --- | --- | --- |
| Face | |  |  |  |
|  | Width of the face | 0.24 | 0.17 | 0.99 |
|  | Width of the mandible | 0.23 | 0.16 | 0.99 |
|  | Height of the face | 0.23 | 0.16 | 0.99 |
|  | Height of the upper face | 0.27 | 0.18 | 0.99 |
|  | Height of the lower face | 0.21 | 0.14 | 0.99 |
|  | Height of the mandible | 0.25 | 0.17 | 0.99 |
|  | Height of the chin | 0.25 | 0.18 | 0.99 |
|  | Height of the lower profile | 0.27 | 0.18 | 0.99 |
|  | Height of the midface | 0.25 | 0.17 | 0.99 |
|  | Lower half of the craniofacial height (left) | 0.27 | 0.19 | 0.99 |
|  | Lower half of the craniofacial height (right) | 0.27 | 0.19 | 0.99 |
|  | Mentocervical angle | 0.42 | 0.26 | 0.99 |
|  | Angle of facial convexity | 0.44 | 0.29 | 0.99 |
|  | Angle of total facial convexity | 0.40 | 0.25 | 0.99 |
|  | Angle of the medium facial third | 0.25 | 0.20 | 0.99 |
|  | Angle of the inferior facial third | 0.20 | 0.15 | 0.99 |
|  | Inclination of general profile line | 0.21 | 0.16 | 0.99 |
|  | Inclination of upper face profile line | 0.30 | 0.23 | 0.99 |
|  | Inclination of lower face profile line | 0.31 | 0.19 | 0.99 |
|  | Inclination of lower third face line | 0.83 | 0.57 | 0.99 |
|  | Inclination of the chin | 1.86 | 1.26 | 0.96 |
|  | Facial index | 0.21 | 0.15 | 0.99 |
|  | Mandible-face width index | 0.28 | 0.22 | 0.99 |
|  | Upper face index | 0.22 | 0.17 | 0.99 |
|  | Mandible width-face height index | 0.27 | 0.17 | 0.99 |
|  | Mandibular index | 0.29 | 0.20 | 0.99 |
|  | Upper face-face height index | 0.21 | 0.15 | 0.98 |
|  | Lower face-face height index | 0.27 | 0.21 | 0.98 |
|  | Chin-face height index | 0.21 | 0.16 | 0.99 |
|  | Chin-mandible height index | 0.46 | 0.31 | 0.98 |
|  | Chin index | 1.92 | 1.28 | 0.98 |
|  | Mandibulo-face height index | 0.21 | 0.15 | 0.98 |
|  | Mandibulo-upper face height index | 0.53 | 0.38 | 0.98 |
|  | Mandibulo-lower face height index | 0.37 | 0.28 | 0.96 |
|  | Mandible width-lower third face depth index | 0.22 | 0.14 | 0.99 |
|  | Upper face height-upper third face depth index | 0.68 | 0.47 | 0.90 |

| **S3 Table. Reliability of photogrammetric measurements (continued).** | | | | |
| --- | --- | --- | --- | --- |
| Measurement | | ME | MME | ICC |
|  | Mandible height-lower third face depth index | 0.67 | 0.46 | 0.79 |
|  | Upper-middle third face depth index | 0.24 | 0.15 | 0.99 |
|  | Middle-lower third face depth index | 0.29 | 0.21 | 0.99 |
|  | Upper cheek-upper third face depth index | 0.25 | 0.17 | 0.98 |
| Orbits | |  |  |  |
|  | Intercanthal width | 0.31 | 0.24 | 0.98 |
|  | Biocular width | 0.27 | 0.17 | 0.99 |
|  | Length of the eye fissure (left) | 0.24 | 0.16 | 0.94 |
|  | Length of the eye fissure (right) | 0.24 | 0.17 | 0.96 |
|  | Height of the eye fissure (left) | 0.25 | 0.16 | 0.91 |
|  | Height of the eye fissure (right) | 0.30 | 0.22 | 0.93 |
|  | Intercanthal index | 0.29 | 0.18 | 0.97 |
|  | Orbital width index | 0.93 | 0.60 | 0.95 |
|  | Eye fissure index | 0.95 | 0.60 | 0.89 |
| Nose | |  |  |  |
|  | Width of the nose | 0.24 | 0.16 | 0.98 |
|  | Height of the nose | 0.24 | 0.16 | 0.99 |
|  | Length of the nasal bridge | 0.22 | 0.15 | 0.99 |
|  | Nasal protrusion | 0.26 | 0.17 | 0.95 |
|  | Nasofrontal angle | 1.21 | 0.86 | 0.95 |
|  | Nasal tip angle | 1.98 | 1.26 | 0.84 |
|  | Nasolabial angle | 2.19 | 1.43 | 0.93 |
|  | Nasofacial angle | 0.43 | 0.31 | 0.97 |
|  | Nasomental angle | 0.49 | 0.31 | 0.98 |
|  | Inclination of nasal bridge | 0.39 | 0.27 | 0.99 |
|  | Nasal index | 0.60 | 0.44 | 0.98 |
|  | Nostril-nose width index | 0.79 | 0.50 | 0.95 |
|  | Nostril width-nose height index | 0.48 | 0.30 | 0.97 |
|  | Nasal tip protrusion-width index | 0.79 | 0.56 | 0.93 |
|  | Nasal tip protrusion-nostril floor width index | 1.24 | 0.77 | 0.96 |
|  | Nasal tip protrusion-nose height index | 0.51 | 0.34 | 0.94 |
|  | Nasal bridge index | 0.44 | 0.29 | 0.96 |
| Lips and mouth | |  |  |  |
|  | Width of the philtrum | 0.21 | 0.12 | 0.96 |
|  | Width of the mouth | 0.23 | 0.16 | 0.99 |
|  | Height of the upper lip | 0.25 | 0.18 | 0.97 |
|  | Height of the cutaneous upper lip | 0.22 | 0.14 | 0.98 |
|  | Vermilion height of the upper lip | 0.28 | 0.20 | 0.97 |
|  | Vermilion height of the lower lip | 0.22 | 0.14 | 0.97 |
| **S3 Table. Reliability of photogrammetric measurements (continued).** | | | | |
| Measurement | | ME | MME | ICC |
|  | Height of the cutaneous lower lip | 0.29 | 0.21 | 0.98 |
|  | Height of the lower lip | 0.30 | 0.22 | 0.98 |
|  | Labiomental angle | 2.43 | 1.62 | 0.94 |
|  | Inclination of upper lip | 0.96 | 0.64 | 0.97 |
|  | Inclination of lower lip | 1.12 | 0.75 | 0.97 |
|  | Upper lip height-mouth width index | 0.64 | 0.45 | 0.99 |
|  | Mouth width contour index | 0.25 | 0.23 | 0.96 |
|  | Philtrum-mouth width index | 0.46 | 0.26 | 0.96 |
|  | Medial-lateral cutaneous upper lip height index | 2.44 | 1.88 | 0.86 |
|  | Vermilion-total upper lip height index | 0.71 | 0.44 | 0.98 |
|  | Vermilion height index | 3.55 | 2.24 | 0.94 |
|  | Upper vermilion contour index | 0.41 | 0.34 | 0.99 |
|  | Lower vermilion contour index | 0.33 | 0.23 | 0.99 |
|  | Lower-upper lip height index | 1.59 | 1.02 | 0.92 |
|  | Cutaneous lower-upper lip height index | 2.29 | 1.48 | 0.97 |
|  | Vermilion-total lower lip height index | 0.98 | 0.65 | 0.98 |
|  | Vermilion arch index | 0.58 | 0.44 | 0.99 |
| Cross-regional | |  |  |  |
|  | Upper face height-biocular width index | 0.38 | 0.27 | 0.99 |
|  | Biocular-face width index | 0.27 | 0.21 | 0.99 |
|  | Intercanthal width-upper face height index | 0.39 | 0.27 | 0.98 |
|  | Intercanthal-nasal width index | 0.78 | 0.49 | 0.97 |
|  | Intercanthal-mouth width index | 0.57 | 0.40 | 0.98 |
|  | Nose-face width index | 0.26 | 0.21 | 0.97 |
|  | Nose-mouth width index | 0.62 | 0.36 | 0.97 |
|  | Nose height-face width index | 0.28 | 0.20 | 0.99 |
|  | Nose-face height index | 0.26 | 0.21 | 0.99 |
|  | Nose-upper face height index | 0.27 | 0.17 | 0.97 |
|  | Nose-lower face height index | 0.51 | 0.33 | 0.99 |
|  | Nasal tip protrusion-upper lip height index | 1.48 | 0.90 | 0.89 |
|  | Mouth-face width index | 0.27 | 0.22 | 0.99 |
|  | Upper lip-upper face height index | 0.30 | 0.20 | 0.96 |
|  | Upper lip-mandible height index | 0.88 | 0.66 | 0.95 |
|  | Upper lip-nose height index | 0.63 | 0.43 | 0.96 |
|  | Lower lip-face height index | 0.34 | 0.22 | 0.97 |
|  | Lower lip-mandible height index | 0.46 | 0.31 | 0.98 |
|  | Lower lip-chin height index | 1.48 | 1.04 | 0.98 |

ME$=$method error; MME $=$method of moments; ICC $=$intraclass correlation coefficient. ME and MME were described in millimeters for linear measurements, degrees for angular measurements and profile inclinations, and percentage values for proportion indices.
